# Eosinophils Suppress the Migration of T Cells Into the Brain of *Plasmodium berghei*-Infected *Ifnar1^-/-^* Mice and Protect Them From Experimental Cerebral Malaria

**DOI:** 10.3389/fimmu.2021.711876

**Published:** 2021-09-30

**Authors:** Johanna F. Scheunemann, Julia J. Reichwald, Patricia Jebett Korir, Janina M. Kuehlwein, Lea-Marie Jenster, Christiane Hammerschmidt-Kamper, Matthew D. Lewis, Katrin Klocke, Max Borsche, Kim E. Schwendt, Camille Soun, Stephanie Thiebes, Andreas Limmer, Daniel R. Engel, Ann-Kristin Mueller, Achim Hoerauf, Marc P. Hübner, Beatrix Schumak

**Affiliations:** ^1^ Institute for Medical Microbiology, Immunology and Parasitology, University Hospital Bonn, Bonn, Germany; ^2^ Parasitology Unit, Centre for Infectious Diseases, Heidelberg University Hospital, Heidelberg, Germany; ^3^ Institute for Experimental Immunology and Imaging, University Hospital Essen, Essen, Germany; ^4^ Clinic for Anesthesiology and Intensive Care, University Hospital Essen, Essen, Germany; ^5^ German Center for Infection Research (DZIF), Heidelberg, Germany; ^6^ German Center for Infection Research (DZIF), Partner Site Bonn-Cologne, Bonn, Germany

**Keywords:** experimental cerebral malaria (ECM), IFNAR1, malaria, CD8 T cells, eosinophils, *Plasmodium berghei*, NK cells, CCL5

## Abstract

Cerebral malaria is a potentially lethal disease, which is caused by excessive inflammatory responses to *Plasmodium* parasites. Here we use a newly developed transgenic *Plasmodium berghei* ANKA (*PbA_Ama1_OVA*) parasite that can be used to study parasite-specific T cell responses. Our present study demonstrates that *Ifnar1^-/-^* mice, which lack type I interferon receptor-dependent signaling, are protected from experimental cerebral malaria (ECM) when infected with this novel parasite. Although CD8^+^ T cell responses generated in the spleen are essential for the development of ECM, we measured comparable parasite-specific cytotoxic T cell responses in ECM-protected *Ifnar1^-/-^* mice and wild type mice suffering from ECM. Importantly, CD8^+^ T cells were increased in the spleens of ECM-protected *Ifnar1^-/-^* mice and the blood-brain-barrier remained intact. This was associated with elevated splenic levels of CCL5, a T cell and eosinophil chemotactic chemokine, which was mainly produced by eosinophils, and an increase in eosinophil numbers. Depletion of eosinophils enhanced CD8^+^ T cell infiltration into the brain and increased ECM induction in *PbA_Ama1_OVA*-infected *Ifnar1^-/-^* mice. However, eosinophil-depletion did not reduce the CD8^+^ T cell population in the spleen or reduce splenic CCL5 concentrations. Our study demonstrates that eosinophils impact CD8^+^ T cell migration and proliferation during *PbA_Ama1_OVA*-infection in *Ifnar1^-/-^* mice and thereby are contributing to the protection from ECM.

## Introduction

Despite major elimination efforts, malaria is still one of the ten leading causes of death in developing countries, with over 400,000 annual deaths (1). *Plasmodium falciparum* is responsible for over 99% of malaria cases in Africa and can lead to serious complications such as cerebral malaria (CM) (1). This complication is mainly caused by inflammatory immune responses of the infected host to parasite-specific components and toxins (2, 3). Several experimental models are used to investigate the immune responses towards parasites such as infection of C57BL/6 mice with *Plasmodium berghei* ANKA. This model mirrors the situation in CM patients to a reasonable extent, including the breakdown of the blood-brain-barrier, and allows the study of *Plasmodium* blood-stage infection in a complete homeostatic system (2, 4–6). Several factors, like interferon gamma (IFN-γ), have been linked to severity of malaria in both mice and humans (7–12) and a fundamental role in the etiology of experimental cerebral malaria (ECM) has also been attributed to T cells activated in the spleen (4, 5, 13–17). However, IFN-γ and T cells are also important for parasite control. Furthermore, IFN-γ production at an early time point of infection has been correlated with protection in mice and humans, as macrophages activated by IFN-γ show enhanced phagocytosis to remove parasites (16, 18–21). Thus, the immune system has to maintain the balance of generating adequate immune responses against the parasite and preventing self-inflicted damage (16, 21).

The contribution of pro-inflammatory mediators, such as type I interferons (IFNs), in the immunopathology and regulation of immune responses towards infection with *Plasmodium* parasites is of major interest (22). The induction of type I IFNs upon *Plasmodium* infection in general (23) and *P. berghei* infection in particular was demonstrated already about 50 years ago and subsequently also in human malaria (4, 24–28). The *in vivo* relevance of type I IFNs during *Plasmodium* infection can be studied in mice that genetically lack a functional type I IFN receptor. This model allows to identify type I IFN-dependent protective mechanisms, which else is challenging due to the lack of reliable type I IFN-depletion methods. As with any genetically modified organism, immunological side effects have to be considered. Studies in genetically modified type I IFN receptor deficient mice have shown that type I IFNs aid protection by inhibiting parasite development but at the same time suppress humoral immunity during *Plasmodium-*infection, as well as T cell activation (29–32). CD4^+^ T cells are important for the differentiation of CD8^+^ effector T cells and shaping the humoral immune response and depletion experiments suggest their contribution to ECM (14, 33). However, no direct effector role was associated with disease onset on day 6 post-infection (p.i.) (34).

Type I IFNs are induced early upon *Plasmodium* infection (32) and *Ifnar1^-/-^* mice have been described to be at least partially protected from ECM upon infection with wild type (WT) or transgenic *P. berghei* ANKA parasites (24, 26, 28, 35, 36). Since ECM pathology is dependent on CD8^+^ T cells and type I IFNs are capable of providing signal three for T cell activation (37), we first addressed here whether *Ifnar1^-/-^* mice benefit from reduced cytotoxic T cell (CTL) activation in the spleen and in consequence from abrogated ECM.

To elucidate antigen-specific T cell responses and their activity *in vivo*, we generated a transgenic *PbA_Ama1_OVA* parasite that expresses a truncated C-terminal fragment of OVA under a blood-stage specific promoter of the apical membrane antigen-1 (AMA-1) gene, a merozoite surface antigen of the parasite. Using the resulting *PbA_Ama1_OVA* clonal line, we demonstrate here that ECM protection in *PbA_Ama1_OVA-*infected *Ifnar1^-/-^* mice was not due to an altered antigen-specific cytotoxic CD8^+^ T cell activity in comparison to infected WT mice. However, infected *Ifnar1^-/-^* mice retained more T cells in the spleen, concomitantly with an induction of large numbers of eosinophils. Importantly, eosinophil depletion reduced the protection of *Ifnar1^-/-^* mice from ECM and led to an increased CD8^+^ T cell number in the brain.

Thus, we present a so far unrecognized role of type I IFN signaling in combination with eosinophils in ECM pathology. We conclude that the protection of *PbA_Ama1_OVA*-infected *Ifnar1^-;/-^* mice from ECM was not based on impaired CD8^+^ T cell generation, but was due to an alternative or regulatory milieu in the periphery – substantially mediated by eosinophils - that prevented the migration of CD8^+^ T cells into the brain in the absence of type I IFN signaling.

## Material and Methods

### Animals

For all experiments, six-week-old female C57BL/6 mice or NMRI (Naval Medical Research Institute) mice were purchased from Janvier Labs, Saint-Berthevin, France, and kept under specified pathogen-free conditions within the animal facility at Heidelberg University (IBF) or IMMIP, University Hospital Bonn. *Ifnar1^-/-^* mice (38) were bred in the House of Experimental Therapy (HET), University Hospital Bonn. All animal experiments were performed according to European regulations and approved by the state authorities (AZ 84-02.04.2012.A264 and AZ 81-02.04.2019.A083, Landesamt für Natur, Umwelt und Verbraucherschutz, Recklinghausen, Germany). Water and food were provided *ad libitum*. Animals were checked daily for wellbeing and scores 0-20 were used for the grading of disease burden according to the RMCBS (39).

### Generation of OVA-Expressing *P. berghei* ANKA Parasites

Transgenic OVA-parasites (*PbA_Ama1_OVA*) were generated to express ovalbumin-derived CD8 and CD4 T cell epitopes at distinct stages of their life cycle (**Figure S1**). Thus, the sequences encoding the SIINFEKL (OVA MHC class I) and TEWTSSNVMEERKIK (OVA MHC class II) peptide sequences were cloned under the control of the promoter and the corresponding signal peptide of AMA1. The gene targeting construct was based on a derivative of b3D.DT^H.^D namely b3D+, both containing *Toxoplasma gondii* DHFR/TS as a selectable marker for resistance to pyrimethamine (40).

For transfection of *P. berghei* schizont-enriched parasites, the targeting plasmid was linearized and afterwards transfected into PbGFPcon parasites by electroporation using the Nucleofactor® solution and Amaxa device (Lonza, Wuppertal, Germany) as described earlier (41). Transfected parasites were injected intravenously into naïve NMRI mice, and selected by providing pyrimethamine in the drinking water. For obtaining clonal parasite populations, limited dilution series with one parasite per defined volume were injected intravenously into 10 naïve recipient NMRI mice. Genotyping of WT and recombinant parasites was performed by PCR using parasite genomic DNA with primer combinations (**Table S1**).

### *Anopheles* Infection Cycle

The transgenic blood-stage OVA parasite (*PbA_Ama1_OVA*) was tested for its life cycle progression in order to rule out potential growth impairment due to the insertion of ovalbumin into the parasite’s genome. To this end transgenic blood-stage OVA-parasites were allowed to grow in NMRI mice for several days until they have reached a high proportion of sexual stages (gametocytes). Gametocytemic mice were then anesthetized and fed to five to seven days-old female *Anopheles* mosquitoes for 15 to 20 min. Mosquitoes were kept at 80% humidity and 21-24°C. Vital life-cycle progression of the transgenic parasite was then compared to infections with WT, i.e. sporozoites were dissected from mosquito midguts on day 14 and from mosquito salivary glands on day 17 to 21 after blood meal. 10,000 infectious sporozoites isolated from salivary glands were additionally used for intravenous infection of inbred C57BL/6 mice to test the capacity of the transgenic parasite line to normally infect the mammalian host i. e. to complete the clinically silent intrahepatic phase and initiate the pathological blood-stage infection. To this end infected mice were tested for parasitized erythrocytes by daily blood smears and analyzed after Giemsa staining and light microscopy. The parasites behave indistinguishable from WT parasites throughout the malarial life cycle.

### Parasites, Infection, and Disease

*Plasmodium berghei* transgenic parasites (*PbA_Ama1_OVA*) containing MHC class I restricted epitopes from chicken ovalbumin were generated as described above. Frozen stabilates of the parasites were stored in liquid N_2_. Stock mice received 200 µL of the stabilates i. p.. After 4 days blood smears were performed to determine the parasitemia of the stock mice (see below). For experimental infections, 5x10^4^ red blood cells infected with *PbA_Ama1_OVA* (iRBC) were then injected intravenously (i. v.) in 100 µL PBS into the mice. From d+3 p. i., the mice were analyzed for symptoms related to ECM with the help of the recently described health score called rapid murine coma and behavior scale (RMCBS) (39). Briefly, the scoring bases on ten different parameters that evaluate health and behavior (gait, balance, motor performance, body position, limb strength, touch escape, pinna reflex, toe pinch, aggression, grooming). Each parameter is rated individually with a maximum of two points (good response) to zero (no response). Due to ethical reasons and in agreement with our ethical license, animals with a score of six or less were sacrificed. This humane endpoint was also applied for the “survival experiment”. For the survival experiment, 10 mice per group were infected with *PbA_Ama1_OVA* and monitored, dependent on their RMCBS score, until day 15 p.i.

### Parasitemia Determination

Blood was collected from the tail vein and thin blood smears were prepared. The blood slides were air dried and fixed in 100% methanol. Giemsa staining solution was prepared by diluting Giemsa stock (Merck KGaA, Darmstadt Germany) 1:20 in Giemsa buffer pH 7.2. Slides were stained for 20 mins followed by rinsing in tap water for 1 min, after which they were left to airdry. Approximately 1000 red blood cells were counted, the infected cells noted and the percentage of iRBCs calculated [=(iRBCs/counted RBCs)*100].

### Evans Blue Assay for Determination of Blood-Brain-Barrier Stability

On d+6p.i. mice received 200 μL of 2% Evans Blue (Sigma-Aldrich, St. Louis, MO, USA) in 0.95% NaCl i. v.. One hour later, mice were sacrificed and the brains were removed, photographed and weighed, thereafter they were placed in 15 mL tubes containing 2 mL formamide (Sigma-Aldrich, St. Louis, MO, USA) and incubated for 48 h at 37°C. Quantification of Evans Blue extravasation was performed by transferring 100 μL of the incubated formamide/brain solution in 96 well plates in triplicates. The standard curve was prepared by diluting Evans Blue to 200 μg/mL with 0.95% NaCl (starting concentration), and diluted 1:2 in duplicates with formamide. The concentration of Evans Blue was measured spectro-photometrically at 620 nm. The amount of infiltrated dye was calculated as μg Evans Blue/g brain tissue x2.

### Harvesting and Preparation of Organs and Flow Cytometric Analysis

Blood was collected from the *Vena fascialis* and the mice were afterwards anaesthetized with 4:1 Ketamine-Rompun mixture (0.01 mL/10 g body weight). After intracardial perfusion (1 X PBS for 5 minutes), spleens and brains were isolated, cut into small pieces, and digested with 0.5 mg/mL collagenase VIII (Roche, Basel, Switzerland) at 37 °C for 30 min. The tissues were then gently passed through a metal sieve (Ø 70 µm), single cells were washed with 1X PBS containing 1% FCS and 2 mM EDTA. Leukocytes from the brain were enriched as described before with a Percoll gradient (42). Cells were counted using CASY^®^ TT Cell counter and then adjusted to 1x10^7^ cells/ml. For analysis by flow cytometry, 1x10^6^ cells were stained in 1X PBS containing 1% FCS and Rat IgG (diluted 1:1000, Sigma-Aldrich, St. Louis, MO, USA) for 20 min on ice. Intracellular staining was performed using the eBioscience™ Foxp3/Transcription Factor Staining Buffer Set (eBioscience, San Diego, CA, USA), according to the manufacturer’s protocol. To detect SIINFEKL-specific CD8^+^ T cells in spleens of *PbA_Ama1_OVA*-infected mice, 1x10^6^ splenocytes were incubated in 30 μL FACS buffer (1X PBS containing 1% FCS) containing 2.5 μL Rat IgG and 5 μL SIINFEKL-H-2K^b^ pentamers coupled to allophycocyanin (ProImmune, Oxford, UK) for 20 min at room temperature. Next, 20 μL FACS buffer containing diluted fluorescently labeled anti-CD8a and CD11a antibodies were added and incubated for another 15 min on ice. Then, the cells were washed with FACS buffer and stored on ice until analysis. For intracellular staining of IFN-γ, CCL5 and Granzyme B (GrzB) the cells were treated with Golgi Plug/Stop (protein transport inhibitors) for at least 4 h to prevent secretion of these effector molecules. For that purpose, 1x10^6^ cells per sample were added to a 96-well cell culture plate and centrifuged for 5 min at 4°C and 400 x*g*. The cells were resuspended in 200 µL complete RPMI medium + 0.8 µL/mL Golgi Plug + 0.8 µL/mL Golgi Stop (1 x concentrated) and cultured for 4 h at 37°C and 5% CO_2_. For IFN-γ the cells were additionally stimulated with 50ng/mL PMA (Invivogen, San Diego, CA, USA), 250 ng/mL ionomycin (Sigma Aldrich, St. Louis, MO, USA) during this 4 h. Afterwards the cells were fixed, permeabilized and stained according to the eBioscience protocol mentioned above. The following fluorescently-labelled specific antibodies used for flow cytometric analysis were purchased from BioLegend, San Diego, CA, USA if not stated otherwise: anti-mouse CD3 (PE-Cy7, BV510 clone 145 2C11), CD4 (Al488, BV605, clone GK1.5), CD8α (BV785, PerCP-Cy5.5, clone 53-6.7), CD11a (Al488, clone M17/4), CD11b (BV510, clone M1-70), CD45 (FITC, PE, PerCP-Cy5.5 clone 30F11), CD49a (PE, clone HMa1), CD49b (Al488, clone HMa2), CD90.2=Thy1.2 (PE, clone 53-2.1), CCL5 (PE, clone 2E9/CCL5), CCR5=CD195 (Al647, clone HM-CCR5), CCR7=CD197 (PerCP-Cy5.5, clone 4B12), CXCR3 (PE, clone CXCR3-173), F4/80 (APC, clone CI:A3-1, BioRad), GATA-3 (PE-Cy7, clone L50-823), GrzB (APC clone GB11, Invitrogen), Lineage cocktail (Pacific Blue, including anti-CD3 clone 17A2, -Ly6C/Ly6G clone RB6-8C5, -CD11b clone M1/70, -CD45R clone RA3-6B2, -TER-119 clone Ter-119, Ly6C (APC-Cy7, clone HK1.4), Ly6G (PE, PE-Cy7, clone 1A8), NK1.1 (BV421, APC-Cy7, clone PK136), NKp46=CD335 (PE-Cy7, clone 29A1.4) RELM-α purified polyclonal, rabbit, (PeproTech, Inc., Rocky Hill, NJ, USA) in combination with goat anti-rabbit Al488 (Invitrogen, Carlsbad, CA, USA), Siglec-F (PE, clone E50-2440, BD, San Jose, USA), T-bet (APC, clone 4B10), TCRβ (Al700, clone H57-597). Data acquisition was performed on a LSR Fortessa^®^ (BD, San Jose, USA) and CytoFLEX S (Beckman Coulter, Brea, CA, USA) and analyzed with FlowJo^®^ Software V10 (FlowJo, LLC, Ashland, OR, USA).

### Immunohistochemistry of Brain Samples

Organs were fixed with 0.05 M phosphate buffer containing 0.1 M L-lysine (pH 7.4, Roth), 2 mg/mL NaIO4 (Roth), and 10 mg/mL paraformaldehyde (Sigma-Aldrich, St. Louis, MO, USA) overnight at 4°C. Afterwards, the organs were equilibrated in 30% sucrose (Carl Roth, Karlsruhe, Germany) solution for 24 h and then frozen in OCT (Weckert Labortechnik) and stored at −80°C. Consecutive sections (5 μm) were mounted on Super Frost Plus glass slides (R. Langenbrinck), dried for 10 min at 70°C, rehydrated with PBS with 0.05% Triton X-100 (Carl Roth, Karlsruhe, Germany) and blocked for 1 h with PBS containing 1% bovine serum albumin (GE Healthcare, Chicago, IL, USA) and 0.05% Triton X-100. The staining was performed in blocking buffer (200 μL volume per section).

The antibodies (SiglecF (clone E50-2440, Alexa647, BD Biosciences); CD8a (clone 53-6.7, PE, BioLegend) were incubated for 1h, DAPI (2 mg/mL, Life Technologies, diluted 1:5000) was incubated for 5 min. After each staining step, three washing steps of 5 min with 0.05% Triton X-100 in PBS were performed. Sections were imaged with the Zeiss Axio Observer.Z1 and Apotome (Carl Zeiss, Oberkochen, Germany) at the Imaging Center Essen and processed by the ZEN Software (Carl Zeiss, Oberkochen, Germany), ImageJ and R. The image processing was done on ImageJ and R by generating cell- and tissue masks. Masks are images where the background is black (i.e., value 0), and the foreground, the object of interest, is white (i.e. value 1). The foreground signals were used to analyze the cell populations of interest, characterized by their emplacement, area and the distance to a point of interest. Tissue and cell signals were then coregistered to determine the location of individual cells, and cell population densities.

### *In Vivo* Cytotoxicity Assay

The lytic activity of SIINFEKL-specific CD8^+^ T cells in *PbA_Ama1_OVA*-infected animals was determined with an *in vivo* cytotoxicity assay on day 6 p.i. as described before (42). Briefly, splenocytes from syngenic donor mice were pulsed with 1 μM of the ovalbumin–derived specific H-2k^b^ peptide SIINFEKL (PEPscreen**/**Sigma-Aldrich, St. Louis, MO, USA) for 30 min at 37°C and subsequently with 1 μM of 5(6)-Carboxyfluorescein diacetate *N*-succinimidyl ester (CFSE, Sigma-Aldrich, St. Louis, MO, USA) for 15 min (CFSE^high^, specific target cells). Reference cells were not pulsed with peptide and labeled with 0.1 μM CFSE for 15 min (CFSE^low^, reference cells). Cells were washed with 1 X PBS (PAA, Cölbe, Germany) and the cell number was determined. The cell populations were mixed at a 1:1 ratio (CFSE^high^/CFSE^low^). Each recipient received 5x10^6^ cells of each population diluted in 0.9% NaCl (Braun, Melsungen, Germany) into the tail vein on day 5 p.i.. Mice were sacrificed 18 h later on day 6 p.i. and spleens were isolated to prepare single-cell suspensions as described above. Lysis of peptide-loaded cells was quantified by measuring the ratio of CFSE^high^ **/**CFSE^low^ cells *via* flow cytometry (Canto II, BD Biosciences). The percentage of specific lysis, termed S8L-specific lysis, was calculated using the following equation: 100 - [(CFSE^high^/CFSE^low^) _immunized_/(CFSE^high^/CFSE^low^) _naïve_] x 100.

### Depletion of Eosinophils in *Ifnar1^-/-^* Mice

After infection with *PbA_Ama1_OVA*, *Ifnar1^-/-^* mice received 1.5 µg/g body weight anti-Siglec-F (R&D Systems, Minneapolis, MN, USA) i. p. in 100 or 150 μL PBS on day 3, 4 and 5 after infection. Mice were analyzed for symptoms related to ECM according to RMCBS. The depletion of eosinophils was confirmed by flow cytometric analysis of the spleen on day 6 after *PbA_Ama1_OVA* infection.

### Cytokine ELISA

Organs from experimental animals were isolated on day 6 p.i. and single cell suspensions were prepared as described above. 1x10^6^ splenocytes or 1.25x10^6^ cells from brain homogenates were cultured in triplicates in 200 μL RPMI 1640 (Life technologies Corporation, Grand Island, NY, USA) incl. 10% fetal calf serum (FCS) (PAA, Cölbe, Germany) 1% penicillin/streptomycin/L-Glutamin (Life technologies Corporation, Grand Island, NY, USA) medium overnight. The supernatants were analyzed by sandwich ELISA for IL-10, IL-13, IFN-γ or TNF (all eBiosciences), CCL5 and GrzB (DuoSet R&D Systems, Minneapolis, MN, USA) according to the manufacturers’ protocols.

### CD11b Cell Sorting Using Magnetic Beads, Arginase Assay, RNA Purification and qPCR

From digested spleens, single cell suspensions were prepared and 4x10^7^ cells were incubated with anti-CD11b^+^ antibodies coupled to magnetic beads accordingly to the manufacturer’s protocol (Miltenyi, Bergisch Gladbach, Germany). Cell separation was performed with the AutoMACS^®^ (Miltenyi, Bergisch Gladbach, Germany). Eluted cells were counted and stained to check the purity. For the arginase assay, 1x10^6^ cells MACS-sorted CD11b^+^ cells were plated into 96-well plates and centrifuged at 1000 x g at 4°C for 10 min. Cells were then lysed with 100 μL of 10 mM Tris-HCl, pH 7.4 (Sigma-Aldrich, St. Louis, MO, USA), containing 1 μM pepstatin A (Sigma-Aldrich, St. Louis, MO, USA), 1 μM leupeptin (Sigma-Aldrich, St. Louis, MO, USA), and 0.4% (w/v) Triton X-100 (Sigma-Aldrich, St. Louis, MO, USA) for 10 min. Lysates were centrifuged at 14,000 x g at 4 °C for 10 min. Supernatant was used for the arginase assay. Analysis for urea quantity was then performed according to manufacturer’s instructions (Abnova, Taipeh, Taiwan). Arginase activity was calculated as units per liter of sample (U/L).

For RNA purification, MACS-sorted CD11b^+^ cells were homogenized in TRIzol^®^ (Thermo Fisher Scientifics, WA, USA) in BashingBeads tubes (Zymo Research, Freiburg, Germany). PeqGOLD HP Total RNA Kit was used for purification according to the manufacturer’s instructions. Thereafter, on-membrane DNase I digestion (peqGOLD, Erlangen, Germany) was performed. For qPCR, TaqMan^®^ RNA-to-Ct™ 1-Step Kit (Life technologies Corporation, Grand Island, NY USA) was used with 2 μg total RNA in a reaction volume of 20 μL. Each sample was normalized to beta-actin. The expression level was calculated as fold-increase against naïve as described (43).

### Primer Sequences

Primers (Seqlab, Göttingen, Germany) were used: YM1 FWD 5’ gcccttattgagaggagcttta 3’, REV 5’ tacagcagacaagacatcc 3’; Arginase FWD 5’ cctatgtgtcatttgggtgga 3’, REV 5’ caggagaaaggacacaggttg 3’; Beta actin FWD 5’ agagggaaatcgtgcgtgac 3’, REV 5’ caatagtgatgacctggcggt 3’.

### Statistical Analysis

GraphPad Prism software version 8 (GraphPad Software, San Diego, CA, USA) was used for statistical analysis. Mann-Whitney-U-test was used to test for significant differences between two unpaired groups. Kruskal-Wallis test, followed by Dunn’s *post hoc* multiple comparisons was used to test for significant differences between multiple groups. Survival experiments were analyzed with Log-rank (Mantel-Cox) test. If not stated otherwise, values from each individual mouse and median with interquartile ranges (IQR) are shown. p values <0.05 were considered as significant. Prior to pooling data were analyzed for homogeneity by not passing Spearman’s test for heteroscedasticity. If data could not be pooled, but statistical trends (p<0.1) were confirmed by several experiments, the trends were included in the figure.

## Results

### Transgenic *PbA_Ama1_OVA* Parasites Induce ECM in WT Mice Whereas *Ifnar1^-/-^* Mice Are Protected

ECM is the result of strong inflammatory immune responses towards *PbA* parasites in susceptible C57BL/6 mice driven by CD8^+^ T cells and IFN-γ (13, 34, 44). Type I IFNs are induced early upon *Plasmodium* infection (26) and *Ifnar1^-/-^* mice lacking type I IFN signaling have been shown to be protected from ECM (26, 28, 35, 36, 45). Since type I IFNs are also relevant in T cell activation, we investigated whether this protection was due to an impaired antigen-specific cytotoxic activity. To investigate this, we generated a transgenic *PbA_Ama1_OVA* parasite clone constitutively expressing a truncated C-terminal fragment (SIINFEKL) of ovalbumin (OVA) including both CD8^+^ T and CD4^+^ T cell epitopes under the control of the AMA-1 promoter in *PbA*, termed *PbA_Ama1_OVA* (**Figure S1** and **Table S1**). This allows the identification of parasite-specific T cell responses by working with commonly available OVA.

**Figure 1:**
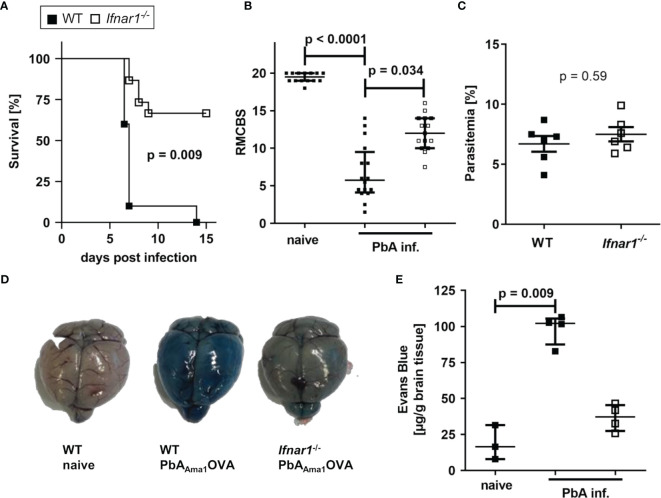
*PbA_Ama1_OVA*-infected *Ifnar1^-/-^* mice are protected from ECM. **(A–E)** C57BL/6 WT and *Ifnar1^-/-^* mice were infected i.v. with 5x10^4^ *PbA_Ama1_OVA*-infected red blood cells and monitored for ECM development and parasitemia. **(A)** Days until onset of ECM, stated as survival of infected mice. Due to ethical reasons animals were sacrificed with a score of six or less. **(B)** Neurological symptoms of *PbA_Ama1_OVA*-infected (PbA inf.) mice according to the rapid murine coma and behavior scale (RMCBS) on day 6 p.i. compared to naïve WT controls. **(C)** Blood-stage parasitemia levels on day 6 p.i. **(D, E)** The stability of the blood brain-barrier was analyzed with an Evans Blue assay on day 6 p.i. in PbA inf. mice and naïve controls. **(D)** Photos from brains isolated from representative animals one hour after injection of Evans Blue. **(E)** Colorimetric quantification of dye extravasation from isolated brains of naïve and PbA inf. mice on day 6 p.i. **(A)** n=10 per group; Log-rank (Mantel-Cox) test. **(B)** n=14-16 per group, data are pooled from 4 individual experiments; Kruskal-Wallis with Dunn’s post-test. **(C)** n=6 per group, representative for 3 individual experiments; Mann-Whitney-U-test. **(E)** n=3-4 per group. Kruskal-Wallis with Dunn’s post-test. **(B, C, E)** Data show median with IQR. p.i., post infection; PbA inf., *PbA_Ama1_OVA*-infected, RMCBS, rapid murine coma and behavior scale.

SIINFEKL-specific CD8^+^CD11a^+^ T cells in the spleens of *PbA_Ama1_OVA*-infected (PbA-infected) WT mice were detected on day 6 p.i., thus confirming the successful generation of endogenous parasite-specific T cells (**Figure S2**). Whereas PbA-infected WT mice succumbed to ECM within 6-7 days, infected *Ifnar1^-/-^* mice showed an increased survival (65%) and less ECM symptoms (**Figures 1A, B**), which is in line with previous reports from studies performed with other *PbA* clones (28, 35, 36, 46). Since parasitemia did not differ significantly between both groups of infected mice (p=0.59), an inefficient parasite replication in infected *Ifnar1^-/-^* mice as a possible reason for ECM protection was excluded (**Figure 1C**). Protection of infected *Ifnar1^-/-^* mice correlated with an intact blood-brain barrier (BBB), whereas the brains of ECM-positive WT mice showed a permeable BBB, demonstrated by an intensive staining due to strong extravasation of the i. v. injected Evans Blue dye (**Figures 1D, E**), also shown previously with another transgenic *P. berghei* ANKA strain (35). Thus, the lack of type I IFN signaling prevented *PbA_Ama1_OVA - *infection-induced ECM in the genetically deficient *Ifnar1^-/-^* mice.

### Less Cellular Infiltrates in Brains of *PbA_Ama1_OVA*-Infected *Ifnar1^-/-^* Mice

**Figure 2:**
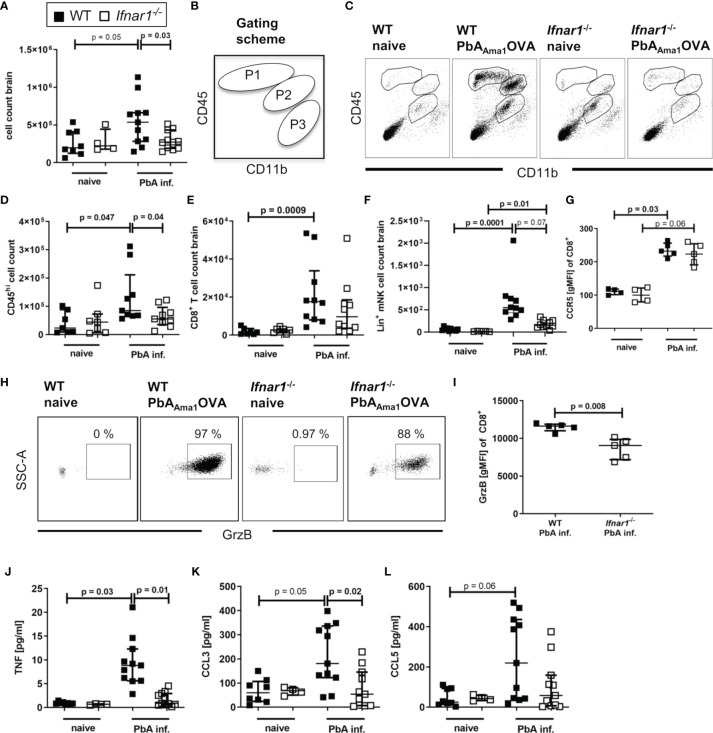
Cellular infiltration and brain inflammation are reduced in *PbA_Ama1_OVA*-infected *Ifnar1^-/-^* mice. **(A–L)** C57BL/6 WT mice and *Ifnar1^-/-^* mice were infected i.v. with 5x10^4^ *PbA_Ama1_OVA*-infected red blood cells and analyzed *ex vivo* on day 6 p.i. regarding cellular brain infiltration *via* flow cytometry and ELISA. **(A)** Enriched brain leukocytes from naïve and infected (PbA inf.) mice were analyzed according to the scheme in **(B)** *via* flow cytometry to distinguish CD11b^lo^CD45^hi^-infiltrated leukocytes (gated in P1) and CD11b^int^CD45^hi^ mononuclear cells (gated in P2) from CD11b^hi^CD45^int^-brain resident immune cells (gate P3). **(C)** Dotplots according to the gating in **(B)** from brain-enriched leukocytes of representative samples. **(D)** Total count of CD45^hi^-infiltrated leukocytes (P1+P2). **(E)** Total count of CD8^+^ T cells (CD45^+^CD3^+^CD8^+^). **(F)** Total count of lineage positive NK cells (CD45^+^Lineage^+^CD49b^+^NKp46^+^T-bet^+^). **(G)** Expression of CCR5 on CD8^+^ T cells. **(H)** Dotplots of Granzyme B (GrzB) expression in CD8^+^ T cells of representative samples. **(I)** Expression of GrzB in CD8^+^ T cells in PbA-inf. mice. **(J–L)** ELISA analysis of enriched brain leukocyte culture supernatants after 24h for **(J)** TNF, **(K)** CCL3 and **(L)** CCL5. **(A–L)** n=4-11, pooled data from 2 individual experiments. **(A, D-G, I–L)** Data show median with IQR. When comparing two groups Mann-Whitney-U-test, for more groups Kruskal-Wallis with Dunn’s post-test. **(A, D)** Mann-Whitney-U-test for comparing the PbA-infected groups. GrzB, Granzyme B; p.i., post infection; PbA inf., *PbA_Ama1_OVA*-infected.

Upon PbA-induced breakdown of the BBB, immune effector cells, parasite-derived particles and soluble mediators can infiltrate the brain. For analysis brains were perfused, digested and associated lymphocytes were enriched by density gradient centrifugation. We observed increased numbers (p=0.05) of infiltrated cells in samples of PbA-infected WT mice compared to WT naïve, but not of PbA-infected and naïve *Ifnar1^-/-^* mice (**Figure 2A**). Further, the number of infiltrated cells was significantly reduced in *Ifnar1^-/-^* compared to WT mice, when considering nominal statistical significance. Flow cytometric analysis of CD11b and CD45 expression allowed for the separation of brain-infiltrating cells (gated in P1 = CD11b^lo^ CD45^hi^ and P2 = CD11b^int^ CD45^hi^) from the periphery *versus* brain-resident microglia (gated in P3 = CD11b^hi^ CD45^int^) (**Figure 2B**). Brain samples of naïve mice and of ECM-protected *Ifnar1^-/-^* mice contained only very few CD45^hi^ cells, whereas brains of PbA-infected WT mice that suffered from ECM had significantly more CD45^hi^ cells (**Figures 2C, D**). Among CD45^hi^ infiltrates, brains of PbA-infected WT mice contained significantly increased frequencies of CD8^+^ T cells (**Figure 2E**) and NK cells (**Figure 2F**) compared to naïve WT mice. These cells were less prominent in brains of PbA-infected *Ifnar1^-/-^* mice, which is in line with data from other *PbA* clones (46). The trend in reduced NK cell numbers between PbA-infected groups was confirmed in three independent experiments, but data did not qualify for pooling all data sets. CD8^+^ T cells from PbA-infected WT mice had significantly increased expression levels of CCR5 (**Figure 2G**) in comparison to naïve WT mice. A relevance for CCR5 in ECM has been previously demonstrated (47, 48). In addition, brain-derived CD8^+^ T cells from infected WT mice expressed Granzyme B (GrzB), thus exhibiting a cytotoxic phenotype (**Figures 2H, I**). However, the few CD8^+^ T cells that were present in brain samples from PbA-infected *Ifnar1^-/-^* mice expressed CCR5 on comparable levels as the PbA-infected WT mice and expressed GrzB (**Figures 2G–I**). In order to analyze another important source for GrzB, we investigated brain-associated NK cells. Interestingly, the GrzB expression in NK cells enriched from brain cell suspensions was significantly higher compared to the expression in CD8^+^ T cells in WT and *Ifnar1^-/-^* mice, suggesting that NK cells might also be involved in the induction of ECM (**Figure S3A**).

Next, we tested whether the production of chemokines and cytokines associated with ECM was altered in brain-associated lymphocyte cultures of PbA-infected *Ifnar1^-/-^* mice after 24h. Whereas samples of naïve controls and infected *Ifnar1^-/-^* mice contained low levels of TNF and CCL3, both mediators were significantly increased in PbA-infected WT mice in comparison to the infected *Ifnar1^-/-^* mice (**Figures 2J, K**). CCL5 levels were low in cultures from naïve cells and enhanced in ~50% of ECM susceptible WT mice following PbA infection (**Figure 2L**). Taken together, these data demonstrate the presence of activated effector cells and molecules in brains of ECM-positive PbA-infected WT mice, whereas lower cell counts and reduced levels of ECM-associated inflammatory mediators indicate only minor brain inflammation in PbA-infected *Ifnar1^-/-^* mice, which correlated with the absence of ECM.

### T Cells Accumulate in Spleens of ECM Protected *Ifnar1^-/-^* Mice With Unaltered Inflammatory and Antigen-Specific CTL Responses

The spleen is essential in the process of immune activation and T cell activation upon encounter with *Plasmodium* parasites.

**Figure 3:**
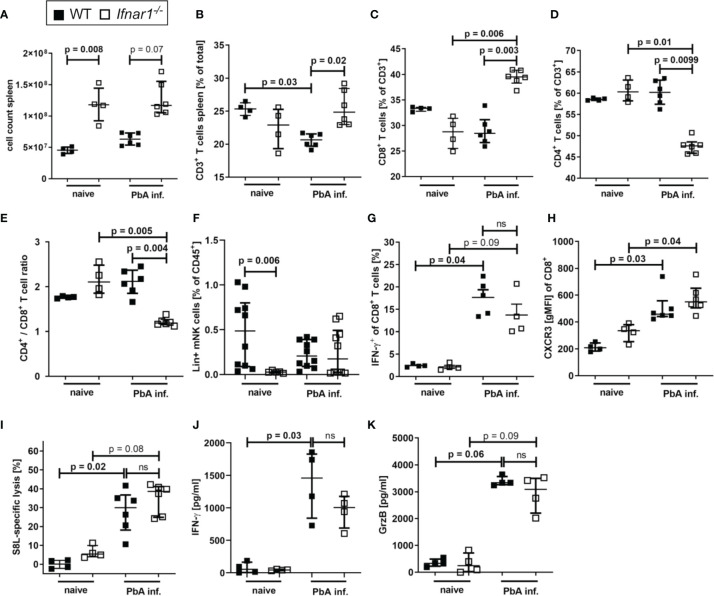
Accumulation of CD8^+^T cell T cells in spleens of *PbA_Ama_OVA*_1_-infected *Ifnar1^-/-^* mice. **(A–K)** C57BL/6 WT mice and *Ifnar1^-/-^* mice were infected i.v. with 5x10^4^ *PbA_Ama1_OVA*-infected red blood cells and analyzed *ex vivo* on day 6 p.i. regarding cellular composition and immune activation in the spleen by flow cytometry **(B–I)** and ELISA **(J, K)**. **(A)** Total splenocyte count. **(B)** Frequency of T cells (CD3^+^) among all splenocytes. **(C)** Frequency of CD8^+^ T cells (CD3^+^CD8^+^) and **(D)** frequency of CD4^+^ T cells (CD3^+^CD4^+^) of all T cells. **(E)** Ratio of CD4^+^ T cells to CD8^+^ T cells in the spleen based on their frequency. **(F)** Frequency of lineage positive NK cells (CD45^+^Lineage^+^CD49b^+^NKp46^+^T-bet^+^) among all splenocytes. **(G)** Frequency of IFN-y positive CD8^+^ T cells among all CD8^+^ T cells after *in vitro* stimulation with PMA and ionomycin. **(H)** CXCR3 on CD8^+^ T cells. **(I)** SIINFEKL (S8L)-specific lysis. **(J, K)** Cytokine quantification in total splenocyte culture supernatants after 24h for **(J)** IFN-γ, and **(K)** Granzyme B (GrzB). **(A–E, H, J, K)** n=4-6, representative for 3 individual experiments **(F)** n= 5-10, WT naïve and PbA-infected data pooled from two individual experiments. **(G, I)** n=4-6, data from 1 experiment. **(A–K)** Data shown as median with IQR; Kruskal-Wallis with Dunn’s post-test. GrzB, Granzyme B; ns, non-significant; p.i., post infection; PbA inf., *PbA_Ama1_OVA*-infected; PMA, phorbol myristate acetate.

In order to further elucidate the events that result in prevention of ECM in *Ifnar1^-/-^* mice, spleens of PbA-infected animals were analyzed on day 6 p.i., the time point of ECM onset in PbA-infected WT mice. The *Ifnar1^-/-^* mice presented a strong splenomegaly, even in the absence of *Plasmodium* infection (**Figure 3A**). Although we observed unaltered parasite-specific cytotoxic functions of splenic T cells in PbA*-*infected *Ifnar1^-/-^* mice in comparison to PbA-infected WT mice (**Figure S2**), the frequency of splenic CD3^+^ T cells differed significantly between the two mouse strains (**Figure 3B**). The T cell frequency was significantly higher in PbA-infected *Ifnar1^-/-^* mice compared to PbA-infected WT mice (p=0.02) (**Figure 3B**). The frequency of CD8^+^ T cells of CD3^+^ T cells increased significantly only in PbA-infected *Ifnar1^-/-^* mice, while the frequency of CD4^+^ T cells of CD3^+^ T cells decreased significantly (**Figures 3C, D**). The observed shift in cell frequencies resulted in a changed CD4^+^/CD8^+^ T cell ratio in the spleen, which was significantly lower in *Ifnar1^-/-^* mice than in WT controls after *PbA_Ama1_OVA* infection (**Figure 3E**). Due to the splenomegaly in *Ifnar1^-/-^* mice, we still observed elevated total splenic CD4^+^ T cell counts in PbA-infected *Ifnar1^-/-^* mice in comparison to infected WT mice (**Figure S4A**). Frequencies of FoxP3^+^ regulatory T cells in PbA-infected *Ifnar1^-/-^* mice were not altered (**Figure S4B**). At the same time we observed increased frequencies of splenic CD4^+^ T cells expressing T-bet, elevated from ~30% in PbA-infected WT mice to ~50% in PbA-infected *Ifnar1^-/-^* mice (**Figure S4C**). Furthermore, spleens of PbA-infected *Ifnar1^-/-^* mice contained a significantly higher percentage of CD4^+^IFN-γ^+^ T cells than spleens of WT controls (**Figure S4D**).

Here this increased frequency of T-bet expressing CD4^+^ was not statistically significant, but increased frequencies of IFNγ^+^ T-bet^+^CD4^+^ T cells have been described in *P. berghei* ANKA-infected *Ifnar1^-/-^* mice before (46).

Since NK cells were detected in the brains of PbA-infected animals and expressed significantly higher levels of GrzB compared to CD8^+^ T cells in the brain (**Figure S3A**), we isolated NK cells from spleens of PbA-infected WT and *Ifnar1^-/-^* mice to examine their activation status. With regard to NK cell frequencies in the spleen (gated according to **Figure S5A**), no differences were observed between *Ifnar1^-/-^* and WT mice after PbA-infection (**Figure 3F**), but splenic NK cells from PbA-infected I*fnar1-/-* mice were less activated (**Figures S3B-E**). In order to investigate whether NK cells were involved in the different outcome of PbA-infection in WT and *Ifnar1^-/-^* mice, NK cells were depleted using anti-NK1.1 antibodies (**Figure S6A** and **S7**). NK cell depletion did not alter ECM development and parasitemia in PbA-infected WT mice (**Figures S6B, C**), which is in line with previous observations (49). Similarly, PbA-infected *Ifnar1^-/-^* mice were still protected from ECM following NK cell depletion (**Figure S6B**), suggesting that differences in NK cell activation were not responsible for the protection of *Ifnar1^-/-^* mice from ECM. In addition, NK cell depleted *Ifnar1^-/-^* mice did not show infiltration of immune cells into the brain (**Figures S6D-G**) and there was still an accumulation of CD8^+^ T cells in the spleens of *Ifnar1^-/-^* mice (**Figures S6H, I**). Furthermore, levels of IFN-γ and GrzB from splenocyte cultures derived from NK cell-depleted *Ifnar1^-/-^* mice were comparable to those of the isotype control treated mice (**Figures S6J, K**). Thus, no changes in the protection from ECM and T cell functions were observed after NK cell depletion, which suggests that NK cells were not essentially involved in the pathogenesis of ECM in WT and *Ifnar1^-/-^* mice.

Splenic CD8^+^ T cells of both WT and *Ifnar1^-/-^* mice neither differed in their IFN-γ production (**Figure 3G**) nor in surface expression of CXCR3 (**Figure 3H**), ICAM or CD11a (**Figure S2**). Since PbA*-*infected *Ifnar1^-/-^* mice had markedly reduced numbers and frequencies of brain infiltrating effector cells – in particular CD8^+^ T cells – we assessed whether this was due to an impaired cytotoxic T cell response upon infection. Transgenic *PbA_Ama1_OVA* parasites successfully induced endogenous SIINFEKL-specific T cells in infected mice (**Figure S2**). The combined capacity of these parasite-induced CTLs to recognize and lyse SIINFEKL-pulsed target cells *in vivo* was comparable in PbA-infected WT and *Ifnar1^-/-^* mice (**Figure 3I**), while the frequency of CD8^+^ T cells in the spleen was higher in PbA-infected *Ifnar1^-/-^* mice compared to PbA-infected WT mice (**Figure 3C**). Similarly, the production of IFN-γ and GrzB by splenocytes from PbA-infected mice was comparable between WT and *Ifnar1^-/-^* mice (**Figures 3J, K**). In summary, our results indicate that T cells were retained in the spleens of PbA*-*infected *Ifnar1^-/-^* mice, and protection of *Ifnar1^-/-^* mice from ECM was not associated with differences in the capability to generate inflammatory and antigen-specific CTL responses. Thus, we hypothesize that primarily the retention of effector T cells in the spleen resulted in protection from ECM and maintained the integrity of the BBB.

### Expansion of CCL5/CCR5 in ECM-Protected *Ifnar1^-/-^* Mice

**Figure 4:**
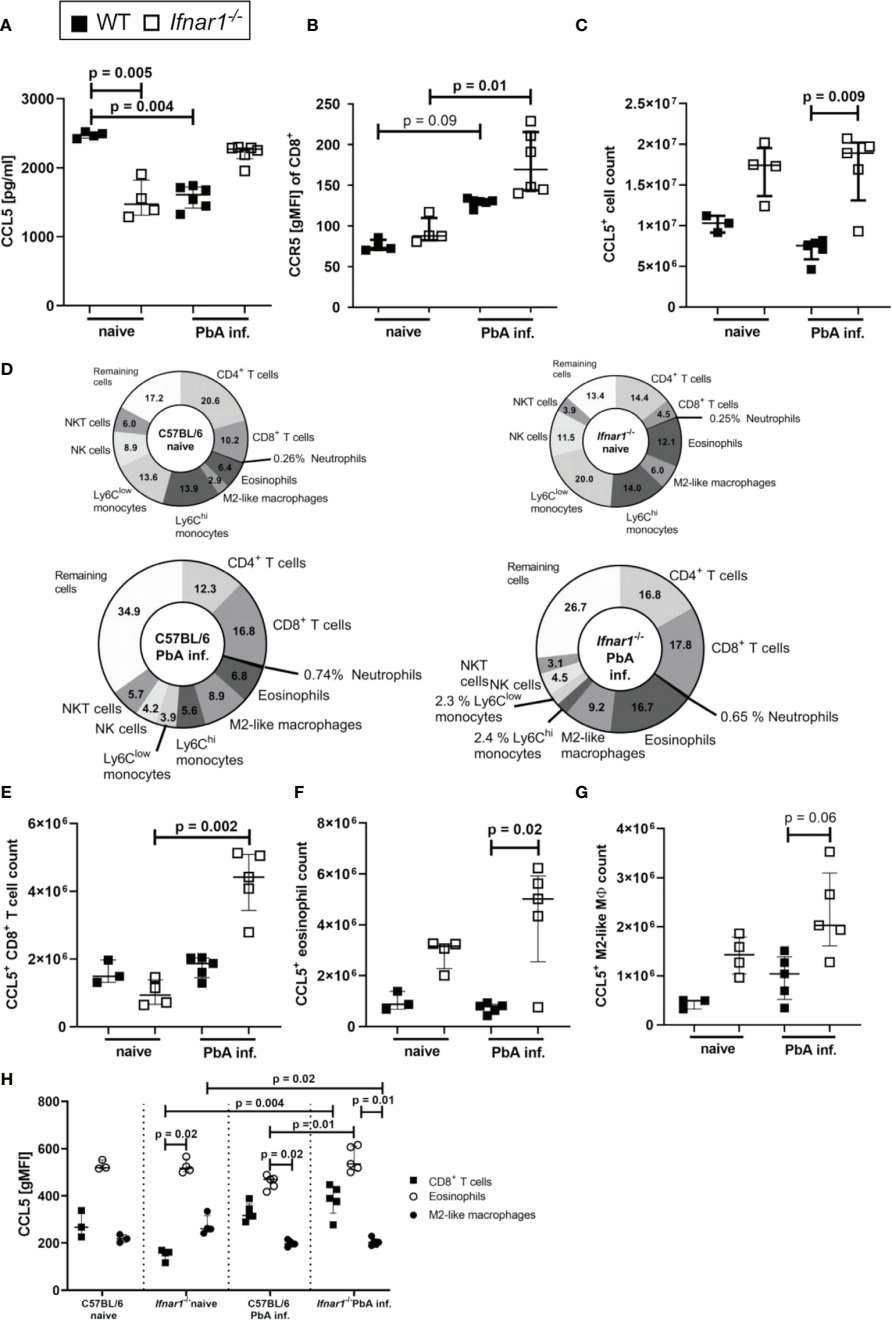
Increased secretion and expression of CCL5/CCR5 in ECM-protected *Ifnar1^-/-^* mice. **(A–H)** C57BL/6 WT mice and *Ifnar1^-/-^* mice were infected i.v. with 5x10^4^ *PbA_Ama1_OVA*-infected red blood cells and spleen cells were analyzed *ex vivo* on day 6 p.i. by ELISA **(A)** and flow cytometry **(B-H)**. **(A)** Cytokine quantification in total splenocyte culture supernatants after 24h for CCL5. **(B)** Expression of CCR5 on CD8^+^ T cells. **(C)** Total count of CCL5 expressing cells in the spleen. **(D)** Overview of CCL5 expressing cell populations in the spleens of naïve and PbA-inf. WT and *Ifnar1^-/-^* mice. **(E)** Total count of CCL5 expressing CD8^+^ T cells (CD3^+^CD8^+^), **(F)** CCL5 expressing eosinophils (CD8^-^CD11b^+^Ly6C^int-hi^Ly6G^-^SiglecF^+^) and **(G)** CCL5 expressing M2-like macrophages (CD11b^+^, Ly6G^-^, SiglecF^-^, F4/80^+^, Relmα^+^). **(H)** CCL5 expression in CD8^+^ T cells, eosinophils and M2-like macrophages. **(A, B)** n=4-6, representative for 3 individual experiments. **(C–H)** n=3-5, data from 1 experiment. **(A–C, E–H)** Data shown as median with IQR; Kruskal-Wallis with Dunn’s post-test. MØ, Macrophage; PbA inf., *PbA_Ama1_OVA*-infected.

Since PbA-infected *Ifnar1^-/-^* mice were protected from ECM despite the successful generation of antigen-specific T cell responses, we analyzed the interaction of CCL5 and CCR5 as one possible factor for mediation of CD8^+^ T cell retention in spleens of *Ifnar1^-/-^* mice. Concentrations of CCL5 were significantly reduced in splenocyte-cultures prepared from PbA-infected WT mice compared to naïve WT mice, while this decrease was not observed upon PbA infection in *Ifnar1^-/-^* mice (**Figure 4A**). CD8^+^ T cells of *Ifnar1* ^-/-^ mice had a significantly higher expression of the CCR5 receptor compared to corresponding naïve controls (**Figure 4B**) and in line with the results on CCL5 release (**Figure 4A**), PbA-infected *Ifnar1* ^-/-^ mice had significantly increased total numbers of CCL5-expressing cells in comparison to PbA-infected WT controls (**Figure 4C**).

In the naïve status the composition of CCL5^+^ cells revealed a higher percentage of CCL5^+^ T cells in WT animals and higher percentages of CCL5^+^ eosinophils and M2-like macrophages in *Ifnar1^-/-^* mice (**Figure 4D**). Upon PbA-infection the proportion of CCL5^+^ monocytes vanished, so that CD8^+^ T cells (17.8%), CD4^+^ T cells (16.8%) and eosinophils (16.7%), together with M2-like macrophages (9.2%) were the main CCL5 producers in spleens of PbA-infected *Ifnar1^-/-^* mice (**Figure 4D**). There was an increase in the CCL5^+^ CD8^+^ T cell count (p=0.002) in *Ifnar1^-/-^* mice upon PbA-infection (**Figure 4E**). The CCL5^+^ eosinophil count was significantly higher (p=0.02) in the spleens of PbA-infected *Ifnar1^-/-^* mice compared to PbA-infected WT mice (**Figure 4F**). Concerning the CCL5^+^ M2-like macrophages, the cell numbers in the spleens of PbA-infected *Ifnar1^-/-^* mice roughly doubled compared to PbA-infected WT mice, but due to the small sample size no statistical significance (p=0.06) was reached (**Figure 4G**). Analysis of the CCL5 expression of each cell population revealed that eosinophils had the highest CCL5 expression in naïve groups of both WT and *Ifnar1^-/-^* mice (**Figure 4H**). Remarkably, eosinophils of PbA-infected *Ifnar1^-/-^* mice expressed significantly more CCL5 (p=0.01) than those of PbA-infected WT mice (**Figure 4H**), which might explain the differences in the splenic CCL5 level between WT and *Ifnar1^-/-^* mice. Taken together, these results suggest a role for eosinophils and the sustained production of CCL5 in the immune response towards PbA-infection in *Ifnar1^-/-^* mice that may contribute to ECM protection.

### Expansion of Type 2-Associated Immune Cells and Accumulation of Eosinophils in Spleens of ECM Negative *Ifnar1^-/-^* Mice

**Figure 5:**
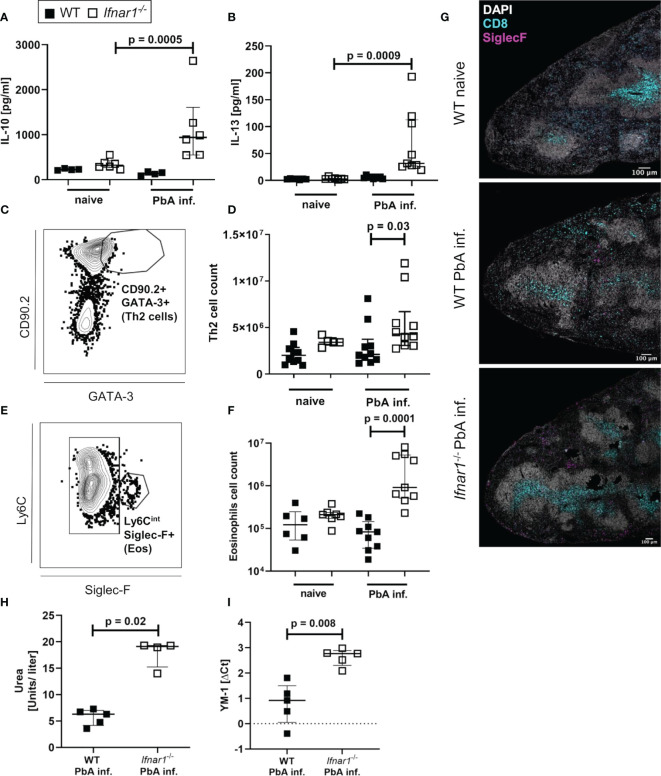
Enhanced type 2-associated immune response in the spleen of *PbA_Ama1_OVA*-infected *Ifnar1^-/-^* mice. **(A–I)** C57BL/6 WT mice and *Ifnar1^-/-^* mice were infected i.v. with 5x10^4^ *PbA_Ama1_OVA*-infected red blood cells and the spleen was analyzed *ex vivo* on day 6 p.i.. Cytokine quantification in total splenocyte culture supernatants after 24h for **(A)** IL-10 and **(B)** IL-13. **(C)** Gating of Th2 cells (gated on CD45^+^Lineage^+^TCRβ^+^cells) and **(D)** total count of Th2 spleen cells. **(E)** Gating of eosinophils (gated on CD8^-^CD11b^+^Ly6G^-^) and **(F)** total count of eosinophils in the spleen. **(G)** Representative immunohistochemical staining of spleen slides of PbA-infected WT and PbA-infected *Ifnar1^-/-^* mice for DNA (DAPI), CD8^+^ T cells (CD8) and eosinophils (SiglecF). Bar in pictures represents 100µm. **(H, I)** MACS-purified CD11b^+^ splenocytes from *PbA_Ama1_OVA*-infected WT *versus Ifnar1^-/-^* mice on day 6 p.i. were analyzed for their **(H)** arginase activity by measuring urea production and expression of **(I)** YM-1 by PCR. Each sample was normalized to β-actin. The expression level was calculated as fold-increase against naïve controls. **(A, B)** n=4-6 per group, representative for 3 independent experiments. **(D, F)** n=5-9, pooled data from 2 individual experiments. **(G)** n=3, data from 1 experiment. **(H)** n=4-5, data representative for 2 individual experiments. **(I)** n=5, data from 1 experiment. **(A, B, D, F, H, I)** Data shown as median with IQR; Kruskal-Wallis with Dunn’s post-test. ΔCt, delta cycle threshold; PbA inf., *PbA_Ama1_OVA*-infected.

After observing similar type 1-associated immune responses in both WT and *Ifnar1^-/-^* mice, but differences in CCL5 expression especially in eosinophils, we analyzed type 2-related cell populations and mediators in *Ifnar1^-/-^* mice and WT controls. The levels of regulatory IL-10 and the type 2-associated cytokine IL-13 were significantly increased in spleen cell cultures of infected *Ifnar1^-/-^* mice compared with cell cultures of uninfected mice (**Figures 5A, B**). This increase was not observed in cultures from infected *vs* uninfected WT mice indicating a general shift towards a type 2-associated immune response in *Ifnar1^-/-^* mice. Moreover, GATA3^+^ type 2 T helper (Th2) cells were analyzed (**Figures 5C, D** and **S5B**) and revealed a significant increase of these cells in the spleens of PbA-infected *Ifnar1^-/-^* mice compared to PbA-infected WT mice (**Figure 5D**). This increase was accompanied by significantly elevated eosinophil counts (**Figures 5E, F** and **Figure S5C**). Immunohistochemical analysis of PbA-infected WT and *Ifnar1^-/-^* spleens confirmed our flow cytometric observations and revealed higher numbers of eosinophils in ECM-protected PbA-infected *Ifnar1^-/-^* mice compared to PbA-infected WT mice, which rarely contained any eosinophils in the spleen (**Figure 5G**). Furthermore, type 2 immune response-related molecules from myeloid cells were increased in PbA-infected *Ifnar1^-/-^* mice, with significantly higher levels of urea, which is obtained after catalyzation of arginine by arginase (**Figure 5H**), as well as significant increases in the expression of YM-1 (**Figure 5I**).

Taken together, these data present a type 2-shifted immune response in ECM-protected *Ifnar1^-/-^* mice and strengthen the hypothesis of a protective role for eosinophils.

### Eosinophils Contribute to ECM Protection in *Ifnar1^-/-^* Mice

After identifying eosinophils as major CCL5 producers and detecting significantly increased eosinophil numbers in the spleens of PbA-infected *Ifnar1^-/-^* mice, we analyzed whether eosinophils were key mediators for ECM protection in PbA-infected *Ifnar1^-/-^* mice.

**Figure 6:**
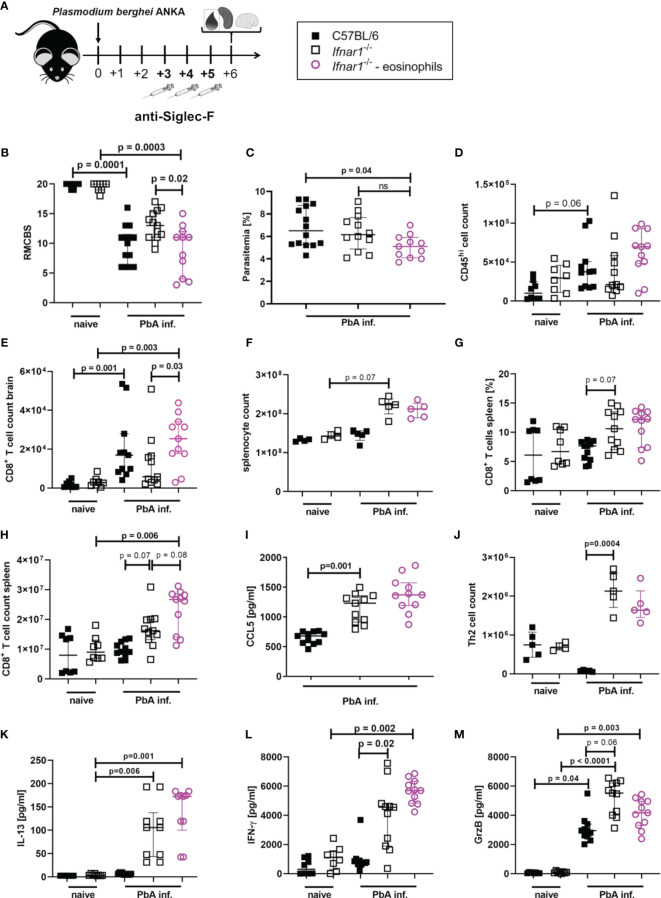
Eosinophil-mediated protection in *PbA_Ama1_OVA*-infected *Ifnar1^-/-^* mice. **(A–M)** C57BL/6 WT mice and *Ifnar1^-/-^* mice were infected i.v. with 5x10^4^ *PbA_Ama1_OVA*-infected red blood cells and a subset of *Ifnar1^-/-^* mice were depleted for eosinophils. **(A)** Experimental setup. **(B)** Neurological symptoms of *PbA*-infected (PbA inf.) mice according to the rapid murine coma and behavior scale (RMCBS) on day 6 p.i. compared to naïve WT controls. **(C)** Blood-stage parasitemia levels determined by Giemsa stains on day 6 p.i.. **(D)** Total count of brain infiltrated CD45^hi^ leukocytes. **(E)** Total count of CD8^+^ T cells (CD3^+^CD8^+^) among infiltrated CD45^hi^-infiltrated leukocytes in the brain. **(F)** Total splenocyte count. **(G)** Frequency of CD8^+^ T cells (CD3^+^CD8^+^) among all splenocytes. **(H)** Total count of CD8^+^ T cells (CD3^+^CD8^+^) in the spleen. **(I)** CCL5 quantification in 24h unstimulated splenocyte culture supernatant. **(J)** Total count of Th2 cells (CD45^+^Lineage^+^TCRβ^+^CD90.2^+^GATA-3^+^) in the spleen. **(K)** IL-13, **(L)** IFN-γ and **(M)** Granzyme B (GrzB) quantification in 24h unstimulated splenocyte culture supernatant. **(B, C)** n=8-14, data pooled from 2 individual experiments. **(D, E, G–I, K–M)** n=8-11, data pooled from 2 individual experiments. **(F, J)** n=4-5, data from one experiment. **(M)** Data shown as median with IQR; Kruskal-Wallis with Dunn’s post-test. **(B, F, G)** Mann-Whitney-U-test between PbA-infected *Ifnar1^-/-^ vs*. eosinophil-depleted *Ifnar1* ^-/-^.GrzB, Granzyme B; ns, non-significant; PbA inf., *PbA_Ama1_OVA*-infected.

Eosinophils were specifically depleted by administration of anti-SiglecF antibodies on day 3, 4 and 5 p.i. with PbA (**Figure 6A** and **Figure S8**), neutrophil counts were not affected. The depletion of eosinophils resulted in a partial loss of ECM protection in PbA-infected *Ifnar1^-/-^* mice with a nominal significant reduction (p=0.02) of RMCBS when comparing PbA-infected *Ifnar1* ^-/-^ mice to depleted *Ifnar1* ^-/-^ (**Figure 6B**). The parasitemia between PbA-infected *Ifnar1* ^-/-^ mice and eosinophil depleted PbA-infected *Ifnar1* ^-/-^ mice was comparable (**Figure 6C**). While PbA-infected *Ifnar1^-/-^* mice remained protected from ECM with scores ranging from 9-17, 50% of the PbA-infected *Ifnar1^-/-^* mice that were specifically depleted of eosinophils reached scores below 8 and were no longer protected from ECM. The drop of RMCBS in *Ifnar1^-/-^* mice after eosinophil depletion was partially based on reduced scores for toe pinch and pinna reflex, which could be related to neuronal malfunction as result of decreased BBB stability. In the brains, the count of CD45^hi^ immune cells and the numbers of CD8^+^ T cells (p=0.03) were increased in the PbA-infected *Ifnar1^-/-^* mice that were depleted of eosinophils as compared to the *Ifnar1^-/-^* mice that had received the isotype control (**Figures 6D, E**). This raised the question whether the eosinophil-mediated protection was due to the retention of CD8^+^ T cells in the spleen. However, the splenomegaly in *Ifnar1^-/-^* mice subsisted in eosinophil depleted mice (**Figure 6F**) and percentages of CD8^+^ T cells in spleens of PbA-infected and eosinophil depleted *Ifnar1^-/-^* mice remained similar to infected *Ifnar1^-/-^* mice that received isotype control antibodies (**Figure 6G**). Furthermore, the count of CD8^+^ T cells was significantly higher in PbA-infected eosinophil-depleted *Ifnar1^-/-^* mice compared to naïve *Ifnar1^-/-^* mice (p=0.006) (**Figure 6H**). The activation of CD8^+^ T cells was not enhanced after eosinophil depletion (**Figure S9**). In line with these observations on CD8^+^ T cell numbers in the spleen, depletion of eosinophils in PbA-infected *Ifnar1^-/-^* did not alter the concentrations of the T cell recruiting chemokine CCL5 in the spleen (**Figure 6I**). Both, eosinophil-depleted and isotype treated *Ifnar1^-/-^* mice had higher levels of CCL5 in the spleens during PbA infection compared to the infected WT controls. Besides eosinophils, Th2 cell counts were significantly increased in the spleens of PbA-infected *Ifnar1^-/-^* mice compared to PbA-infected WT mice (p=0.0004) (**Figure 6J**). Following eosinophil depletion, Th2 cell numbers were still elevated in the PbA-infected *Ifnar1^-/-^* mice, although not statistically significant in comparison to the PbA-infected WT controls. Similarly, levels of the Th2 cytokine IL-13 were significantly increased in spleen cell cultures of PbA infected *Ifnar1^-/-^* mice compared to naïve controls, independent from the eosinophil depletion (**Figure 6K**). Cytokine analysis in the spleen further revealed the highest levels of IFN-γ in PbA-infected *Ifnar1^-/-^* mice depleted of eosinophils (**Figure 6L**). For GrzB the eosinophil depletion had no significant impact when comparing to PbA-infected *Ifnar1^-/-^* mice (**Figure 6M**). Overall, this suggests that the protection from ECM in *Ifnar1^-/-^* mice involves eosinophils by limiting the infiltration of CD8^+^ T cells into the brain, but this seems not to be due to an eosinophil-mediated retention of CD8^+^ T cells in the spleen *via* an enhanced CCL5 secretion.

## Discussion

Immune activation during infection is a double-edged sword for the host. The effector cells and mediators generated to protect the host and eliminate *Plasmodium* parasites require tight control to prevent pathology and the development of CM (21). The interaction of effector immune mechanisms and their regulation during *Plasmodium* infections are still not yet understood in detail. Previous studies correlated genetic variations of *IFNAR1* in African children and ECM models with protection from severe malaria (35, 50). In the present study we demonstrate that eosinophils support the protection of *Ifnar1^-/-^* mice from ECM development. The generation of fully functional parasite-specific cytotoxic T cell responses in the spleen of ECM-protected *Ifnar1^-/-^* mice was not impaired. However, those T cells were retained in the spleen and did not migrate into the brain where they could contribute to ECM. Moreover, spleens of PbA-infected *Ifnar1^-/-^* mice showed a type 2-shifted milieu with significantly increased numbers of eosinophils, and increased levels of the chemoattractant CCL5, which may contribute to the retention of T cells within the spleen.

In this study we used a newly generated transgenic *P. berghei* ANKA parasite clone that stably expresses OVA-derived peptides and causes ECM between day 6 and 9 p.i. in ≥90% of infected WT mice. This is in line with previous studies using other strains of *P. berghei* in WT mice (28, 35, 36). Furthermore, *PbA_Ama1_OVA*-infected *Ifnar1^-/-^* mice showed a survival rate of 65% and comparable parasitemia as seen in infected WT mice, which was similar to the observations by Ball *et al.* and Palomo *et al.*, whereas Edwards *et al.* described a reduced peripheral parasitemia in infected *Ifnar1^-/-^* mice (35, 36, 46). Sharma *et al.* reported a later ECM onset of *P. berghei* ANKA-infected WT mice and 100% protection of infected *Ifnar1^-/-^* mice (28). These inconsistences are likely due to different abilities of *P. berghei* ANKA clones to induce ECM, as well as differences in dose and route of infection used (51).

In mice, ECM pathology has been strongly associated to effector CD8^+^ T cells primed in the spleen that infiltrate the brain (52, 53). The induction of *Plasmodium*-specific T cells in infected WT mice towards antigens from different life cycle stages of *PbA* parasites has been shown before (54, 55). Using *PbA_Ama1_OVA* transgenic parasites expressing ovalbumin-derived peptides during the blood stage, we demonstrate that OVA-specific CD8^+^ T cell responses in infected *Ifnar1^-/-^* mice are comparable to those in infected WT mice. This implies that an efficient priming by dendritic cells occurred, despite the systemic lack of type I IFN signaling in the genetically deficient mice. Accordingly, splenic CD8^+^ T cells in ECM-protected *Ifnar1^-/-^* mice and susceptible WT controls expressed similar levels of IFN-γ. Also, GrzB levels in whole splenocyte cultures were comparable in all infected groups. Both mediators are known to contribute to ECM development (21, 52, 56). These results support findings from other groups (57, 58). Thus, type I IFN signaling is dispensable for the development of CTL responses during *PbA_Ama1_OVA* infection. We conclude that the protection of *Ifnar1^-/-^* mice from ECM was not due to generally impaired or inefficient antigen priming or CTL responses in the spleen, but rather to differences in the T cell recruitment to the brain in the course of PbA infection. Brains of PbA-infected and ECM protected *Ifnar1^-/-^* mice contained lower numbers of recruited CD8^+^ T cells in our study. Such a reduced T cell infiltration into the brains of *Ifnar1^-/-^* mice was previously reported by other studies focusing on type I IFN signaling during *P. berghei* ANKA infection (35, 36). Ball *et al.* showed by elegant transfer experiments that local stimulation *via* the type I IFN receptor on T cells is crucial for T cell accumulation in the brains of infected mice (35), although an altered generation of parasite-specific effector CD8^+^ T cells in the spleen could not be excluded in their study.

T cell influx into the brain during ECM requires CXCR3 and CCR5 (48, 59) and their ligands — CCL3, CCL5, CXCL9 and CXCL10 — are known to be induced in brains of ECM susceptible mice (48, 59, 60). Our study revealed that brains of ECM-protected *Ifnar1^-/-^* mice contained lower levels of CCL3 and CCL5, which may be due to the reduced number of myeloid cells in brains of these mice. In addition to activated T cells, macrophages and monocytes are described as possible sources of CCL5 (61). Interestingly, microglia are an additional source of CCL3 and CCL5 (62) and CCL5 production by microglia from PbA-infected WT mice was suggested to be driven by type I IFN (63). Thus, lower levels of CCL5 in brain samples from PbA-infected *Ifnar1^-/-^* mice could be associated with the genetically disrupted type I IFN signaling. However, CCL5 concentrations and populations of CCL5-positive cells were significantly increased in spleens of PbA-infected *Ifnar1^-/-^* mice, with CD8^+^ T cells, CD4^+^ T cells, eosinophils and M2-like macrophages being the main cell populations expressing CCL5 with eosinophils showing the highest expression. This may result in increased T cell retention in the spleen and thus reduced influx of effector cells into the brain, thereby preventing ECM pathology in *Ifnar1^-/-^* mice.

Besides elevated numbers of CD8^+^ T cells in the brains of ECM positive mice, we also found higher numbers of lineage positive NK cells. Both cell types are potent producers of pro-inflammatory mediators such as IFN-γ and GrzB and are recruited *via* CCL5 (64, 65). However, while depletion of CD8^+^ T cells results in the protection from ECM in WT mice, there are contradictory reports about the effect of NK cell depletion on ECM onset (13, 49, 66, 67), which could be due to differences in the depletion antibodies used with anti-asialo GM1 leading to protection in WT mice in contrast to anti-NK1.1 (13, 66). Apart from that, depletion of NK cells in the ECM model was not performed so far for ECM-protected *Ifnar1* ^-/-^ mice although, NK cells showed a lower activation level in the spleen in these mice suggesting an involvement in the protected phenotype. In our study, similar to the results of the anti-NK1.1 treatment in WT mice, depletion of NK cells *via* anti-NK1.1 had no impact on ECM protection in *Ifnar1^-/-^* mice, as no differences in inflammation and disease severity were observed.

Despite the retention of IFN-γ and GrzB-producing NK and T cells in the spleens of PbA-infected *Ifnar1^-/-^* mice, we detected high levels of the type 2 cytokines IL-10 and IL-13 in spleens of infected *Ifnar1^-/-^* mice. IL-10 is an anti-inflammatory cytokine (68) and may contribute to control the inflammation in *Ifnar1^-/-^* mice. The induction of a type 2 immune response within the spleens of *Ifnar1^-/-^* mice was further highlighted by the increased production of M2-like macrophage-derived urea and YM-1, as well as the presence of eosinophils. Upon PbA infection in WT mice, the immune system shifts towards a type 1 immune response and eosinophils in the spleen disappear. In contrast, the lack of type I interferon signaling resulted in the maintenance of eosinophils. Interestingly, eosinophils, Th2 cells and alternatively activated (M2) macrophage-driven immune responses are commonly induced by helminths and have the potential to downregulate type 1-driven immune responses. Thus, a protection from excessive inflammation in mice lacking type I IFN signaling was associated with the induction of type 2 immune responses that are usually associated with helminth infections. Accordingly, experimental studies described that co-infections with filariae result in the protection from ECM in an IL-10 dependent manner, although the role of eosinophils was not investigated so far (69).

Depletion of eosinophils in PbA-infected *Ifnar1^-/-^* mice confirmed that eosinophils are involved in mediating protection from ECM development, since neurological malfunctions, assessed by the RMCBS, were manifested in depleted animals. Such a potentially protective role of eosinophils was indicated by earlier studies in human patients, as increased eosinophil numbers in the blood were associated with asymptomatic malaria, while eosinophils were reduced during the onset of acute symptoms (70). However, the lower numbers of eosinophils from patients with acute symptoms showed increased activation based on the plasma levels of eosinophil granula proteins. The authors suggest that this eosinophil activation was not part of a Th2-associated immune response and the activation of eosinophils might also contribute to pathogenesis during malaria. Another study linked polymorphisms in the gene for eosinophil cationic protein with the susceptibility to CM (71). Thus, further studies are required to pinpoint the role of eosinophils during the pathogenesis of malaria. In general, it is known that eosinophils are able to exert pro-inflammatory, type 2 immune inducing or even anti-inflammatory effects and the local and systemic immunological milieu may be essential for their protective or detrimental role during malaria.

In our study, *Ifnar1^-/-^* mice showed a generalized type 2 immune dominance that was not reversed after eosinophil depletion, since Th2 cell numbers and IL-13 production by splenocytes remained stable. Similarly, CCL5 levels and CD8^+^ T cell numbers in the spleen remained increased in eosinophil-depleted *Ifnar1^-/-^* mice, suggesting that eosinophils were not essential for the maintenance of type 2 immune responses and the retention of CD8^+^ T cell populations in the spleen in *Ifnar1^-/-^* mice. Nevertheless, the depletion of eosinophils resulted in increased CD8^+^ T cell numbers in the brains of *Ifnar1^-/-^* mice, indicating that eosinophils do protect *Ifnar1^-/-^* mice from ECM by limiting the infiltration of CD8^+^ T cells into the brain by a mechanism that has still to be defined.

Taken together, our data provide important new aspects of type I IFN signaling in the initiation of ECM pathology. We conclude that in PbA-infected *Ifnar1^-/-^* mice functional effector T cells are induced, but their migration into the brain was hampered. This protective effect in *Ifnar1^-/-^* mice was partially dependent on eosinophils, arguably by limiting the proliferation and migration of CD8^+^ T cells into the brain.

## Data Availability Statement

The original contributions presented in the study are included in the article/**Supplementary Material**. Further inquiries can be directed to the corresponding author.

## Ethics Statement

The animal study was reviewed and approved by Landesamt für Natur, Umwelt und Verbraucherschutz, Recklinghausen, Germany.

## Author Contributions

Conceived and designed the experiments: PK, JR, JS, JK, AH, MH, and BS. Performed the experiments: PK, JK, JR, JS, CH-K, ML, MB, KS, KK, DE, CS, and ST. Creation of mutant parasites: CH-K, ML, and A-KM. Analyzed the data: PK, JR, JS, JK, DE, and BS. Contributed reagents/materials/analysis tools: AL, DE, MH, and AH. Wrote the manuscript: PK, JR, JS, MH, and BS. All authors contributed to the article and approved the submitted version.

## Funding

PK was supported by a PhD scholarship from the German Academic Exchange program (DAAD). JR and JS were supported by a PhD scholarship from the Jürgen Manchot Stiftung, Düsseldorf, Germany. JK, JR, JS, and BS were supported by BONFOR 2012-01-22, 2013-01-29, 2017-5-02, 2018-7-01, 2018-7-02, 2019-7-01, 2019-7-02, and 2020-7-03. A-KM is a recipient of a DZIF funded Maternity Leave stipend. AH and BS were members of the Bonn Cluster of Excellence ImmunoSensation EXC 1023 and AH and MH are funded by the Deutsche Forschungsgemeinschaft (DFG, German Research Foundation) under Germany’s Excellence Strategy – EXC2151 – 390873048. AH and MH are members of the German Center for Infection Research (DZIF).

## Conflict of Interest

The authors declare that the research was conducted in the absence of any commercial or financial relationships that could be construed as a potential conflict of interest.

## Publisher’s Note

All claims expressed in this article are solely those of the authors and do not necessarily represent those of their affiliated organizations, or those of the publisher, the editors and the reviewers. Any product that may be evaluated in this article, or claim that may be made by its manufacturer, is not guaranteed or endorsed by the publisher.
