# Supplementary figures and images for: Eosinophils Suppress the Migration of T Cells Into the Brain of Plasmodium berghei-Infected Ifnar1-/- Mice and Protect Them From Experimental Cerebral Malaria
Source: Front Immunol. 2021 Sep 30;12:711876. doi: 10.3389/fimmu.2021.711876 (PMC8514736; doi:10.3389/fimmu.2021.711876)

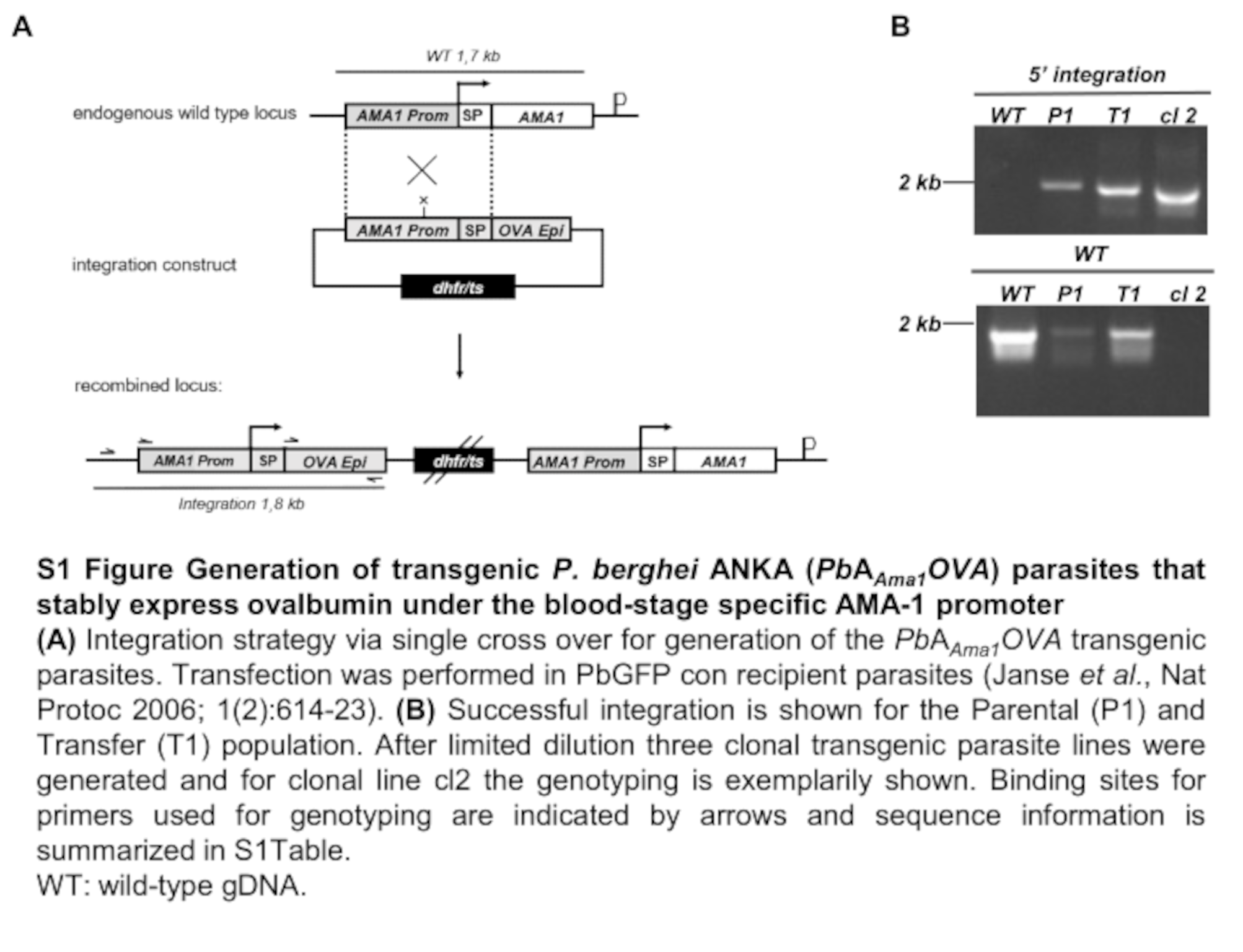

Supplement: Supplementary file 1 [file Image_1.tiff]

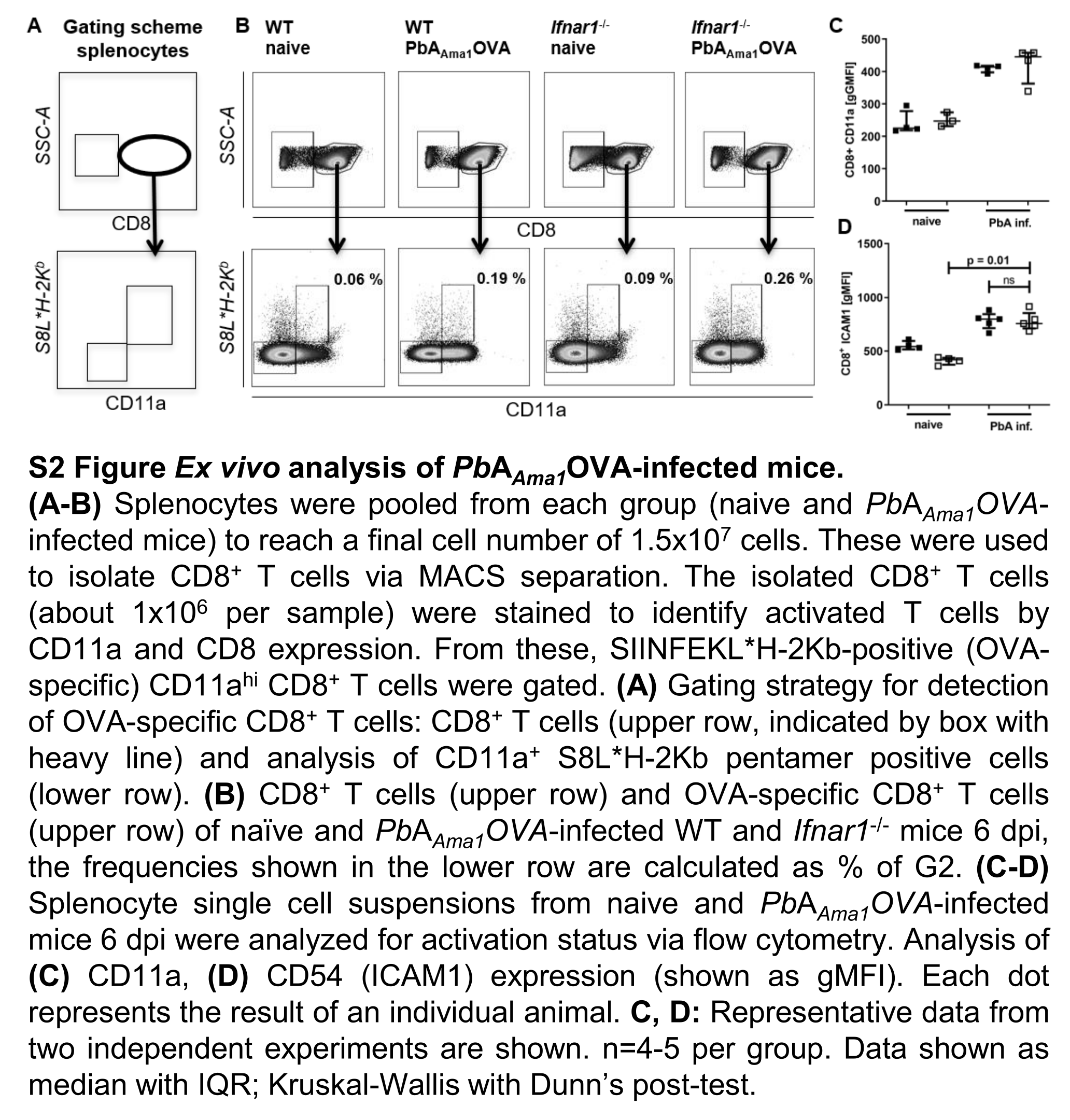

Supplement: Supplementary file 2 [file Image_2.tiff]

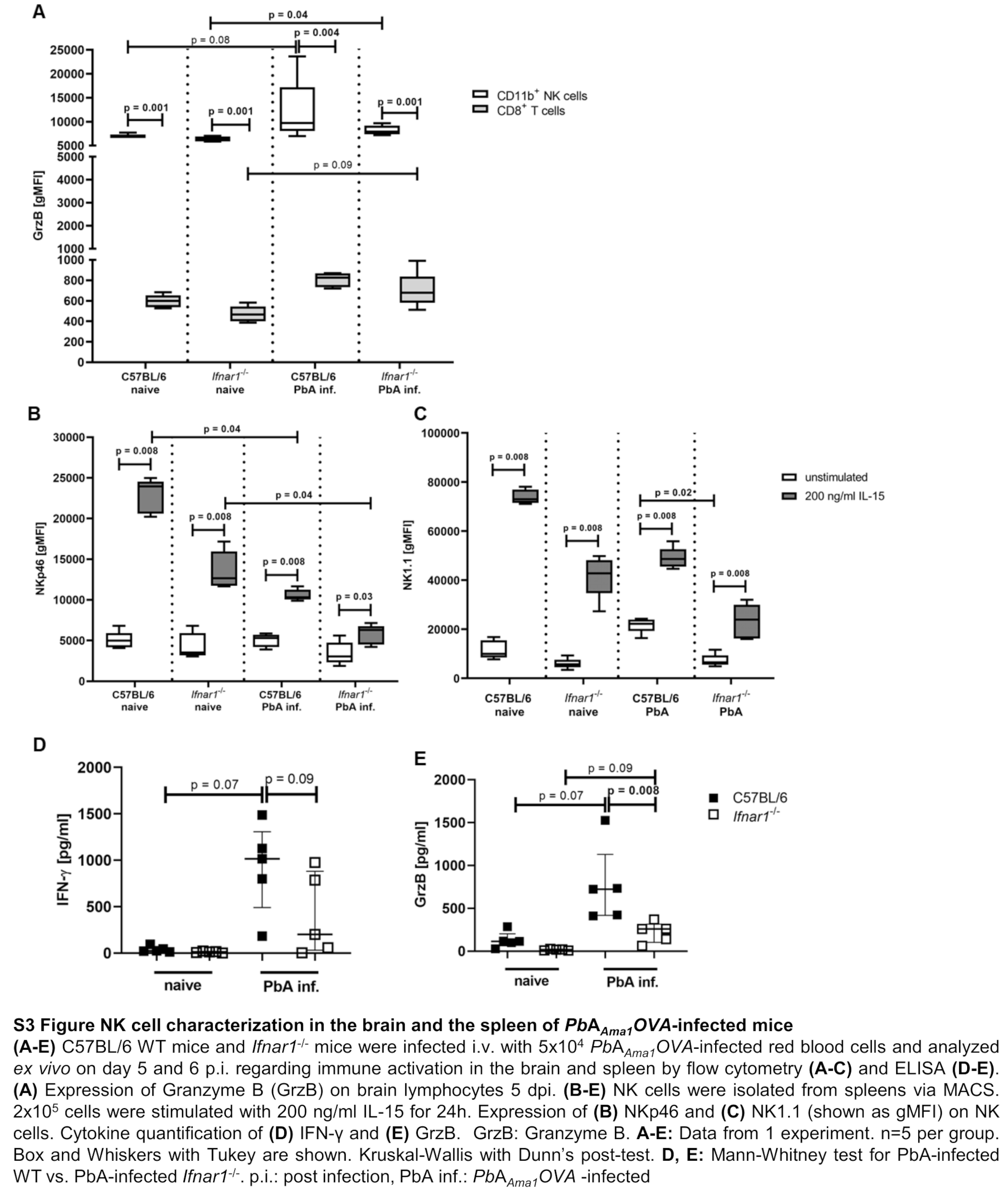

Supplement: Supplementary file 3 [file Image_3.tiff]

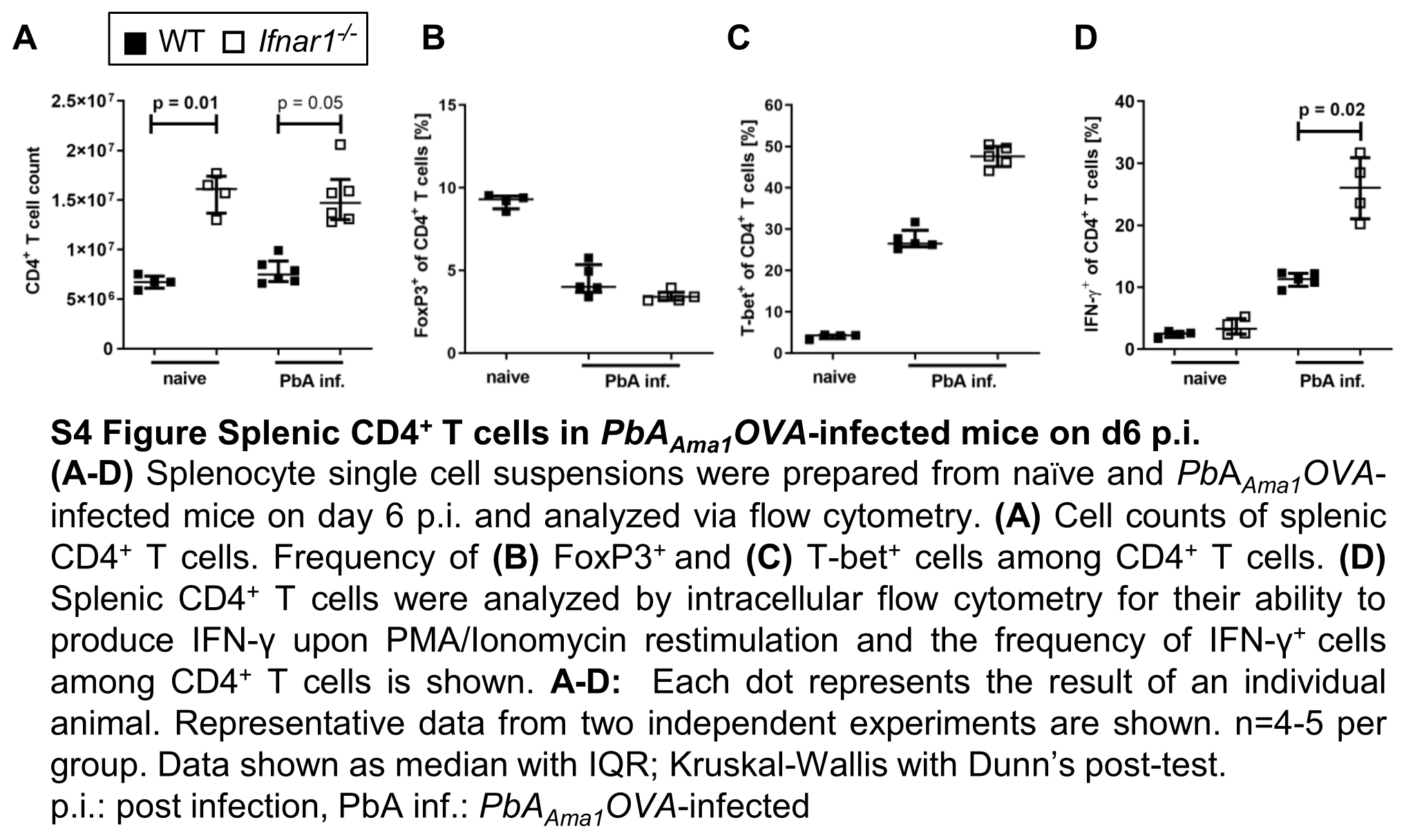

Supplement: Supplementary file 4 [file Image_4.tiff]

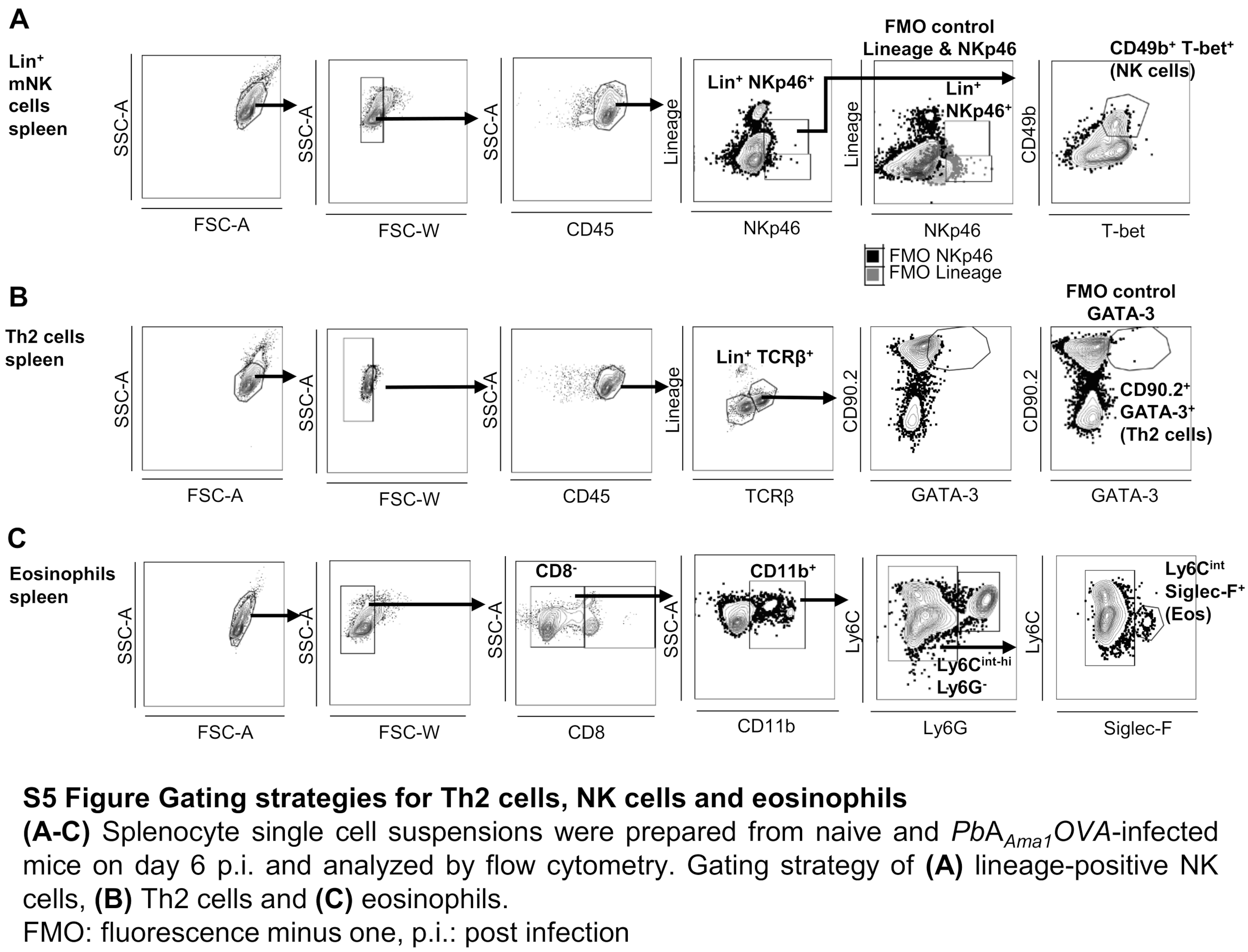

Supplement: Supplementary file 5 [file Image_5.tiff]

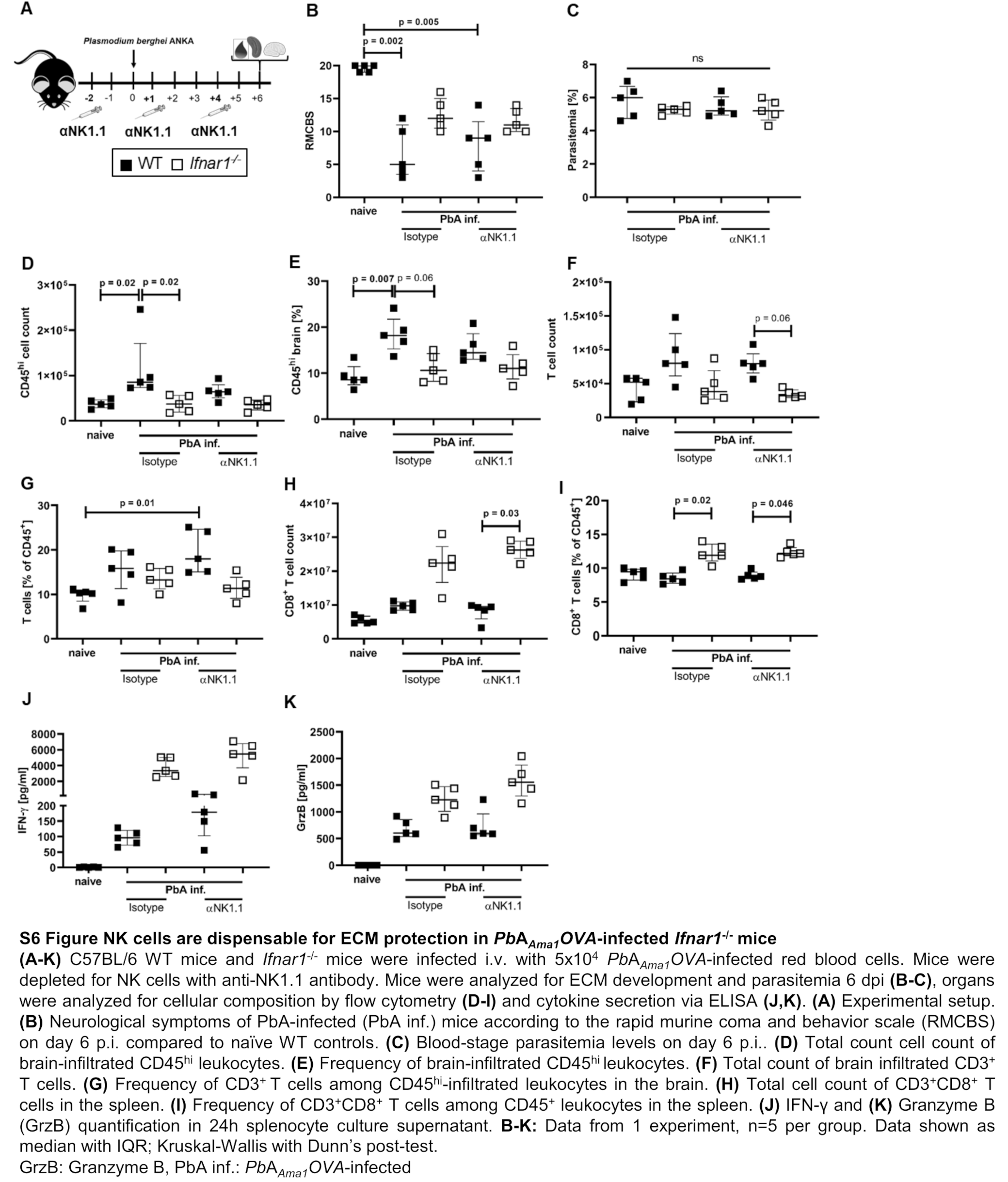

Supplement: Supplementary file 6 [file Image_6.tiff]

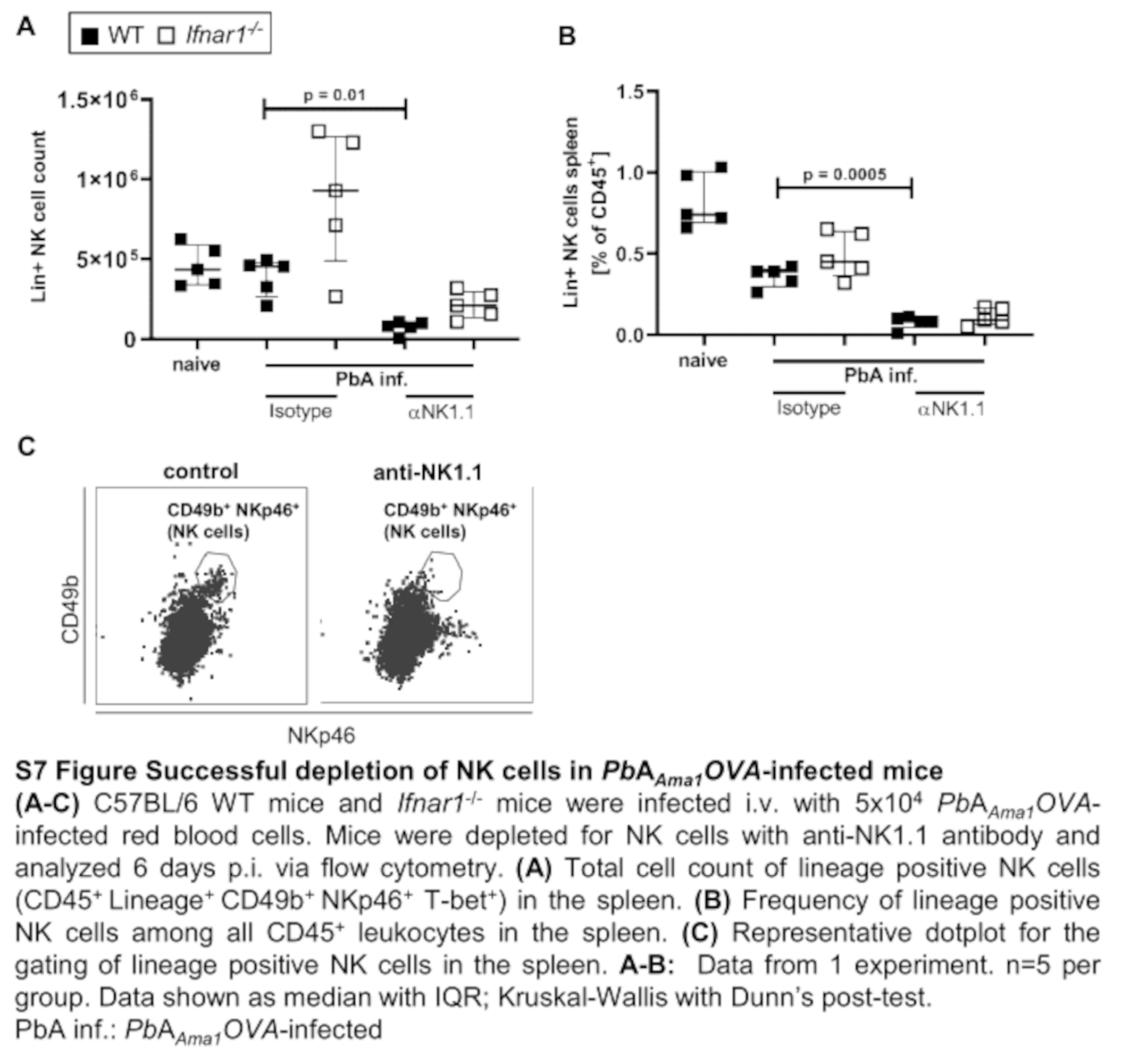

Supplement: Supplementary file 7 [file Image_7.tiff]

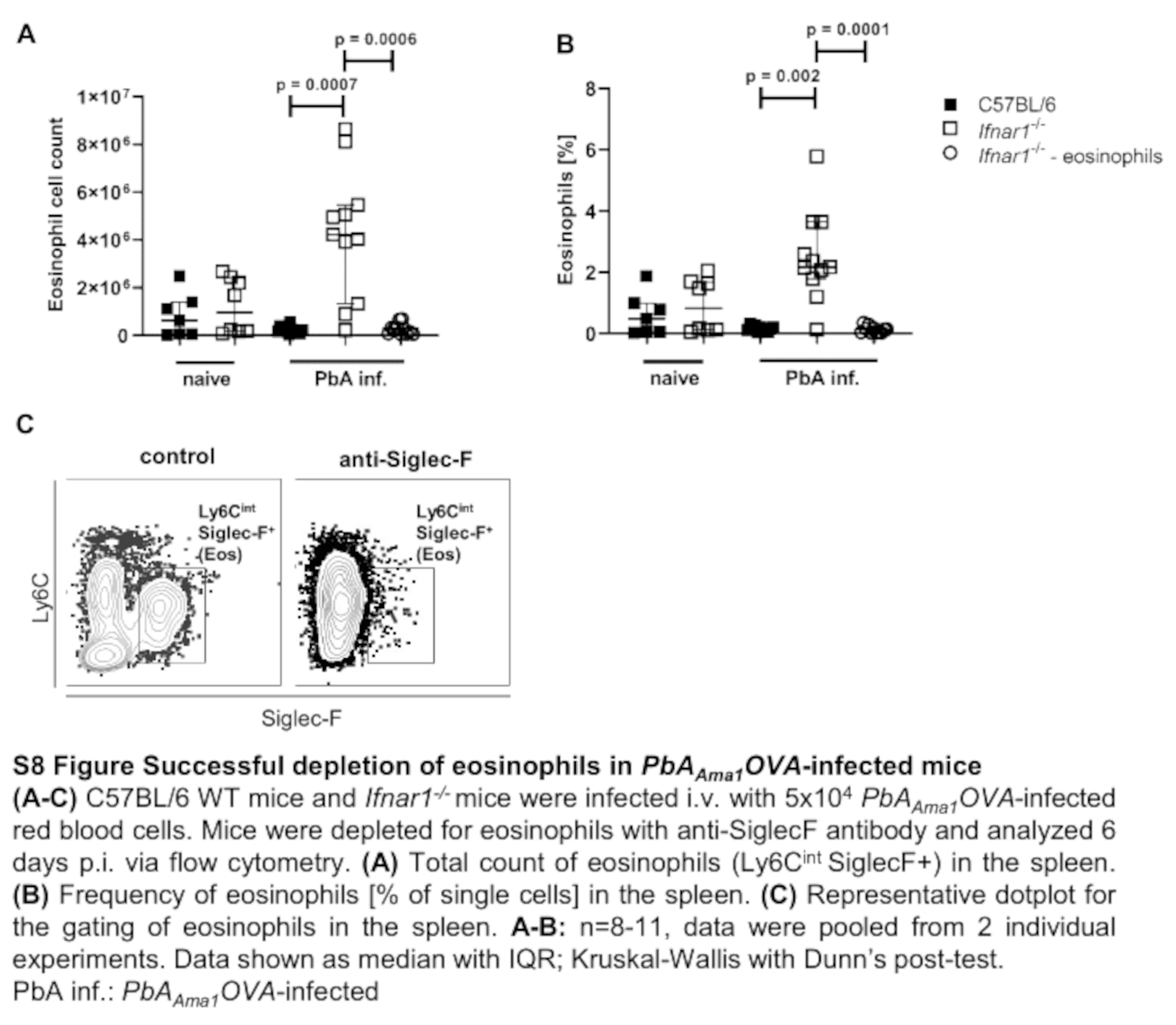

Supplement: Supplementary file 8 [file Image_8.tiff]

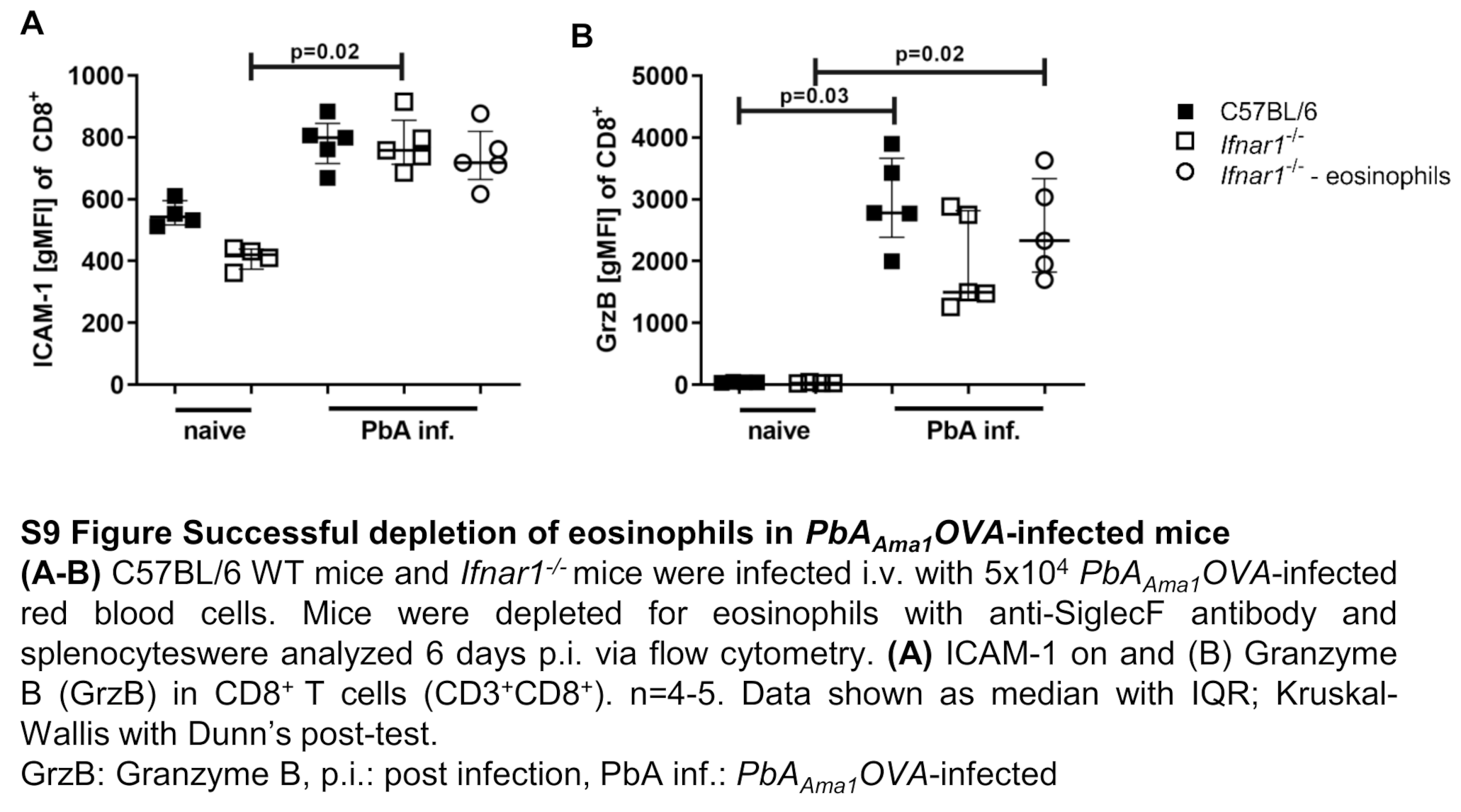

Supplement: Supplementary file 9 [file Image_9.tiff]
