# Supplementary material for: Eosinophils Suppress the Migration of T Cells Into the Brain of Plasmodium berghei-Infected Ifnar1-/- Mice and Protect Them From Experimental Cerebral Malaria
Source: Front Immunol. 2021 Sep 30;12:711876. doi: 10.3389/fimmu.2021.711876 (PMC8514736; doi:10.3389/fimmu.2021.711876)
Supplement: Supplementary file 10 [file Table_1.docx]

**S1 Table List of primers**

Primers were used for both cloning of the targeting construct for transfection and homologous recombination into *P. berghei* GFPcon parasites and respective primer for genotyping.

| **Name** | **Sequence 5'>3'** | **Restriction site** | **Plasmid linearization** | **Notes** |
| --- | --- | --- | --- | --- |
| Pb_AMA1_PromSP_For | CGGGATCCTTAATATATGTAAAAAAAC | *BamHI* |  | cloning of targeting construct |
| Pb_AMA1_PromSP_Rev | GCTCTAGAACAATTGCTCAGGTTTAT | *XbaI* |  | cloning of targeting construct |
| OVA-EPI_XbaI_For | GCTCTAGAGCCGCCATGTTTAGAGTGGCATCAATGGC | *XbaI* | *XbaI* |  |
| OVA-EPI_NotI_Rev | ATTTGCGGCCGCTTAAGGGGAAACACATCTGC | *NotI* |  | Genotyping and cloning |
| Pb_AMA1_test_5' for | TGCGGTTGCAATATAGACAGAAAATATTAAC |  |  | Genotyping |
| Pb_AMA1_test_3'rev | GCTGAATTGGCATTGTTACCGGG |  |  | Genotyping |
